# Impaired dNKAP function drives genome instability and tumorigenic growth in *Drosophila* epithelia

**DOI:** 10.1093/jmcb/mjad078

**Published:** 2023-12-06

**Authors:** Ting Guo, Chen Miao, Zhonghua Liu, Jingwei Duan, Yanbin Ma, Xiao Zhang, Weiwei Yang, Maoguang Xue, Qiannan Deng, Pengfei Guo, Yongmei Xi, Xiaohang Yang, Xun Huang, Wanzhong Ge

**Affiliations:** Division of Human Reproduction and Developmental Genetics, Women's Hospital, Zhejiang University School of Medicine, Hangzhou 310058, China; Institute of Genetics, Zhejiang University School of Medicine, Hangzhou 310058, China; Zhejiang Provincial Key Laboratory of Precision Diagnosis and Therapy for Major Gynecological Diseases, Women's Hospital, Zhejiang University School of Medicine, Hangzhou 310006, China; Division of Human Reproduction and Developmental Genetics, Women's Hospital, Zhejiang University School of Medicine, Hangzhou 310058, China; Institute of Genetics, Zhejiang University School of Medicine, Hangzhou 310058, China; Zhejiang Provincial Key Laboratory of Precision Diagnosis and Therapy for Major Gynecological Diseases, Women's Hospital, Zhejiang University School of Medicine, Hangzhou 310006, China; State Key Laboratory of Molecular Developmental Biology, Institute of Genetics and Developmental Biology, Chinese Academy of Sciences, Beijing 100101, China; Division of Human Reproduction and Developmental Genetics, Women's Hospital, Zhejiang University School of Medicine, Hangzhou 310058, China; Institute of Genetics, Zhejiang University School of Medicine, Hangzhou 310058, China; Zhejiang Provincial Key Laboratory of Precision Diagnosis and Therapy for Major Gynecological Diseases, Women's Hospital, Zhejiang University School of Medicine, Hangzhou 310006, China; Division of Human Reproduction and Developmental Genetics, Women's Hospital, Zhejiang University School of Medicine, Hangzhou 310058, China; Institute of Genetics, Zhejiang University School of Medicine, Hangzhou 310058, China; Zhejiang Provincial Key Laboratory of Precision Diagnosis and Therapy for Major Gynecological Diseases, Women's Hospital, Zhejiang University School of Medicine, Hangzhou 310006, China; Division of Human Reproduction and Developmental Genetics, Women's Hospital, Zhejiang University School of Medicine, Hangzhou 310058, China; Institute of Genetics, Zhejiang University School of Medicine, Hangzhou 310058, China; Division of Human Reproduction and Developmental Genetics, Women's Hospital, Zhejiang University School of Medicine, Hangzhou 310058, China; Institute of Genetics, Zhejiang University School of Medicine, Hangzhou 310058, China; Division of Human Reproduction and Developmental Genetics, Women's Hospital, Zhejiang University School of Medicine, Hangzhou 310058, China; Institute of Genetics, Zhejiang University School of Medicine, Hangzhou 310058, China; Division of Human Reproduction and Developmental Genetics, Women's Hospital, Zhejiang University School of Medicine, Hangzhou 310058, China; Institute of Genetics, Zhejiang University School of Medicine, Hangzhou 310058, China; Division of Human Reproduction and Developmental Genetics, Women's Hospital, Zhejiang University School of Medicine, Hangzhou 310058, China; Institute of Genetics, Zhejiang University School of Medicine, Hangzhou 310058, China; Division of Human Reproduction and Developmental Genetics, Women's Hospital, Zhejiang University School of Medicine, Hangzhou 310058, China; Institute of Genetics, Zhejiang University School of Medicine, Hangzhou 310058, China; Division of Human Reproduction and Developmental Genetics, Women's Hospital, Zhejiang University School of Medicine, Hangzhou 310058, China; Institute of Genetics, Zhejiang University School of Medicine, Hangzhou 310058, China; State Key Laboratory of Molecular Developmental Biology, Institute of Genetics and Developmental Biology, Chinese Academy of Sciences, Beijing 100101, China; Division of Human Reproduction and Developmental Genetics, Women's Hospital, Zhejiang University School of Medicine, Hangzhou 310058, China; Institute of Genetics, Zhejiang University School of Medicine, Hangzhou 310058, China; Zhejiang Provincial Key Laboratory of Precision Diagnosis and Therapy for Major Gynecological Diseases, Women's Hospital, Zhejiang University School of Medicine, Hangzhou 310006, China; Cancer Center, Zhejiang University, Hangzhou 310058, China

**Keywords:** *Drosophila* wing imaginal disc, dNKAP, genome instability, tumorigenic growth

## Abstract

Mutations or dysregulated expression of NF-kappaB-activating protein (NKAP) family genes have been found in human cancers. How NKAP family gene mutations promote tumor initiation and progression remains to be determined. Here, we characterized dNKAP, the *Drosophila* homolog of NKAP, and showed that impaired dNKAP function causes genome instability and tumorigenic growth in a *Drosophila* epithelial tumor model. *dNKAP*-knockdown wing imaginal discs exhibit tumorigenic characteristics, including tissue overgrowth, cell-invasive behavior, abnormal cell polarity, and cell adhesion defects. *dNKAP* knockdown causes both R-loop accumulation and DNA damage, indicating the disruption of genome integrity. Further analysis showed that *dNKAP* knockdown induces c-Jun N-terminal kinase (JNK)-dependent apoptosis and causes aberrant cell proliferation in distinct cell populations. Activation of the Notch and JAK/STAT signaling pathways contributes to the tumorigenic growth of *dNKAP*-knockdown tissues. Furthermore, JNK signaling is essential for *dNKAP* depletion-mediated cell invasion. Transcriptome analysis of *dNKAP*-knockdown tissues confirmed the misregulation of signaling pathways involved in promoting tumorigenesis and revealed abnormal regulation of metabolic pathways. *dNKAP* knockdown and oncogenic Ras, Notch, or Yki mutations show synergies in driving tumorigenesis, further supporting the tumor-suppressive role of dNKAP. In summary, this study demonstrates that dNKAP plays a tumor-suppressive role by preventing genome instability in *Drosophila* epithelia and thus provides novel insights into the roles of human NKAP family genes in tumor initiation and progression.

## Introduction

Recurrent mutations in genes encoding splicing factors have been initially identified in several types of tumors, and these mutations frequently occur in the early stage of disease initiation (Yoshida and Ogawa, 2014; Dvinge et al., 2016). A recent whole-exome sequencing data analysis also revealed that mutations in splicing factor genes are common in cancer (Seiler et al., 2018). Extensive studies focused on the aberrant splicing events mediated by mutant splicing factors have revealed the close relationship between tumor-specific mis-splicing events and transformation (Yoshida and Ogawa, 2014; Anczukow and Krainer, 2016; Dvinge et al., 2016; Sveen et al., 2016). However, genetic evidence for these tumor-specific alternative splicing changes driving tumor initiation and progression is quite limited, and it remains largely unexplored whether other biological processes and signaling pathways affected by mutant splicing factors contribute to tumor initiation and development (Anczukow and Krainer, 2016; Dvinge et al., 2016; Inoue et al., 2016; Sveen et al., 2016; Joshi et al., 2017). Interestingly, several large-scale genetic screens have demonstrated that splicing factors can act as potential regulators of genome instability (Paulsen et al., 2009; Hurov et al., 2010; Adamson et al., 2012). Splicing factors are enriched in the regulators for DNA damage response, raising the possibility that splicing factors can prevent tumorigenesis through a splicing-independent mechanism (Paulsen et al., 2009; Hurov et al., 2010; Adamson et al., 2012; Naro et al., 2015). Thus, understanding the roles of splicing factors in regulating DNA damage, genome instability, and tumor initiation will reveal the potential mechanism underlying tumorigenesis.

Splicing factors comprise hnRNP, SR, and SR-related family proteins (Wahl et al., 2009). Homozygous loss of classical splicing factors often leads to embryonic lethality in mammalian and other systems, making the *in vivo* functional study of these factors more challenging. NF-kappaB-activating protein (NKAP) family proteins are a class of SR-related proteins highly conserved across different species (Burgute et al., 2014, 2016). Genetic and biochemical studies have revealed their important functions in various biological processes, including cell proliferation and apoptosis (Pajerowski et al., 2009, 2010; Hsu et al., 2011; Sanchez-Garcia et al., 2015; Thapa et al., 2016). In humans, there are two NKAP homologues, NKAP and NF-kappaB-activating protein-like (NKAPL) (Burgute et al., 2014). NKAP was first identified to activate NF-kappaB signaling in the nucleus and later shown to function in T cell development as a transcriptional repressor of Notch signaling (Chen et al., 2003; Pajerowski et al., 2009). NKAPL has been shown to be associated with the risk of schizophrenia (Yue et al., 2011). Conditional knockout studies in mice revealed that NKAP is required for adult hematopoietic stem cell maintenance and survival (Pajerowski et al., 2010). NKAP localizes to nuclear speckles and functions in RNA splicing and processing by association with RNA-binding proteins through its RS domain (Burgute et al., 2014). Furthermore, NKAP can bind to various RNAs, including pre-mRNAs and small nuclear RNAs (Burgute et al., 2014). Emerging evidence suggests a link between altered NKAP/NKAPL activity and cancer development. First, mutations in NKAP have been identified in chronic lymphocytic leukemia and endometrial cancer patients, although at a low frequency (Le Gallo and Bell, 2014; Puente et al., 2015; Landau et al., 2017). Second, reduced levels of NKAP expression are found in several types of cancers (Li et al., 2016). Third, NKAP was reported to localize at the kinetochore after being SUMOylated, and SUMOylated NKAP is required for anchoring CENP-E to kinetochores for proper chromosome alignment, the failure of which leads to aneuploidy (Li et al., 2016). Finally, a recent analysis based on human cancer transcriptome data demonstrated that *NKAPL* is a consistently downregulated gene across various cancers (Li et al., 2017). Despite these advances, *in vivo* evidence for NKAP/NKAPL driving tumor initiation and progression remains lacking, and the underlying molecular mechanisms are largely unknown.

*Drosophila* epithelia are an excellent *in vivo* model system for dissecting the molecular mechanism of tumor initiation and progression (Bilder, 2004; Enomoto et al., 2018; Richardson and Portela, 2018). In the present study, we show that the *Drosophila* homologue of NKAP, dNKAP, has a tumor-suppressive activity and prevents genome instability in the wing epithelia.

## Results

### dNKAP exhibits a tumor-suppressive role in Drosophila wing epithelia

*Drosophila dNKAP (CG6066)* encodes a predicted protein of 463 amino acid residues, and the coding region of this gene is intronless (www.flybase.org). A previous large-scale RNAi screen identified *dNKAP* as one of the genes involved in tumor suppression in the *Drosophila* brain (Neumuller et al., 2011). To determine whether *Drosophila* dNKAP has a tumor-suppressive activity and uncover the underlying mechanism, we examined the function of dNKAP in *Drosophila* wing epithelia, a widely used *in vivo* system for studying tumorigenesis.

**Figure 1:**
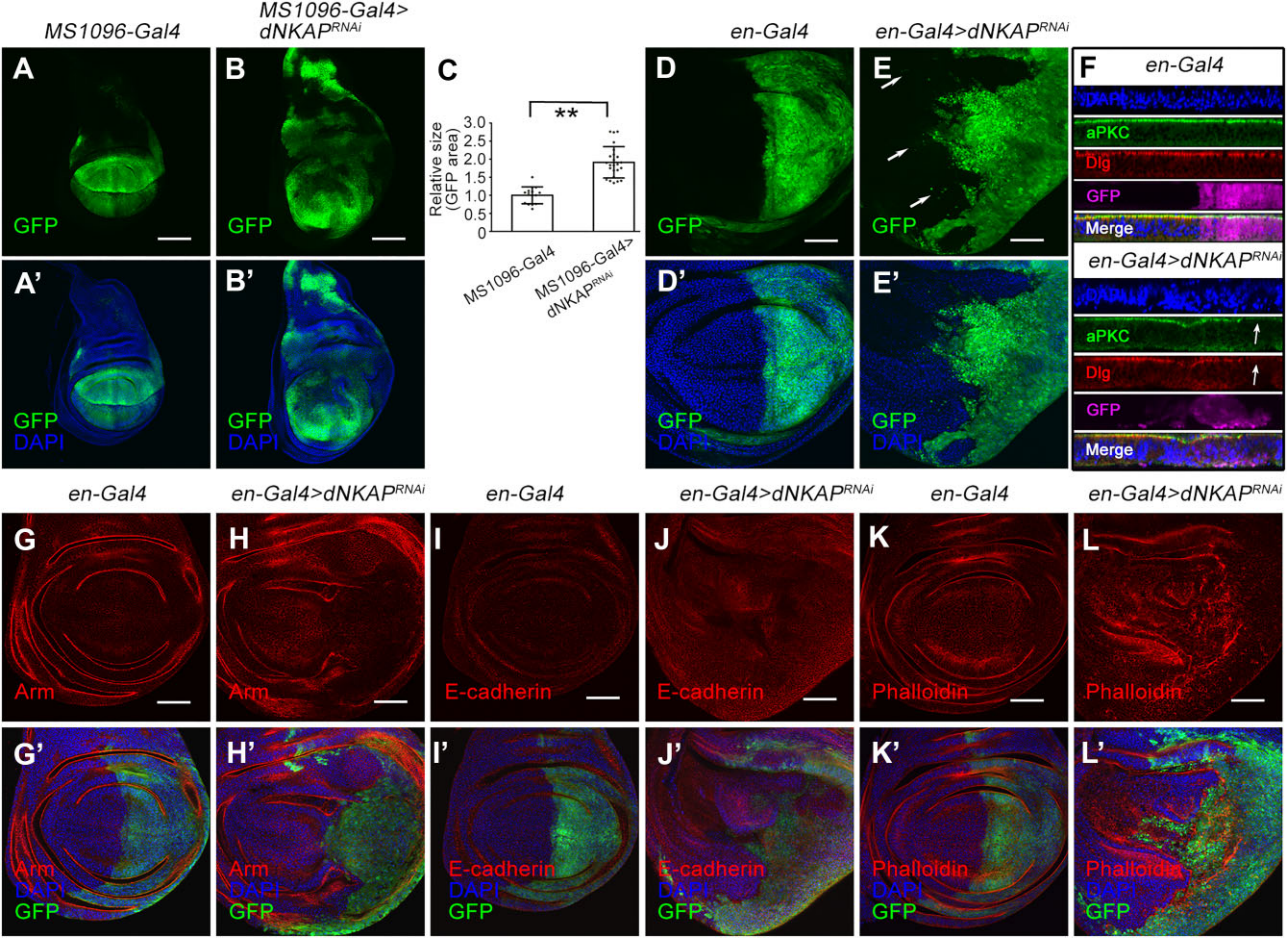
*dNKAP* depletion causes tissue overgrowth with tumorigenic features in the wing imaginal disc. (**A**–**B’**) Increased tissue size in *dNKAP*-knockdown wing discs. Scale bar, 100 μm. (**C**) Quantification of GFP-positive area. The value was calculated by normalizing to the mean area of the control disc. *n = *15 for control and 24 for *dNKAP* RNAi discs. **P < 0.01. (**D**–**E’**) Cell-invasive behavior in *dNKAP*-knockdown wing discs. Note that a few GFP-positive cells migrate toward the anterior compartment (indicated by arrows). Scale bar, 50 μm. (**F**) Disruption of aPKC and Dlg localization in *dNKAP*-knockdown wing discs (indicated by arrows). Shown are optical cross-sections of wing imaginal discs with apical side up. (**G**–**J’**) Reduction in armadillo (Arm) and E-cadherin levels in *dNKAP*-knockdown wing discs. (**K**–**L’**) Accumulation of F-actin in *dNKAP*-knockdown wing discs. Scale bar, 50 μm. GFP was used to mark the knockdown domain. Panels **A**–**B’, D**–**E’**, and **G**–**L’** show single-plane confocal sections of third-instar wing imaginal discs with posterior side to the right and dorsal side up.

We used the GAL4/UAS system to knock down *dNKAP* by RNAi in the *Drosophila* developing wings. The *MS1096-Gal4* driver we used in our study is expressed in the pouch area of the wing disc (along with *UAS-CD8-GFP* marking the expression domain). The previously published *dNKAP* RNAi line was used (Neumuller et al., 2011). *MS1096-Gal4-*driven *dNKAP RNAi* caused a developmental delay of ∼1–2 days and produced viable adults with small crumpled wings (Supplementary Figure S1A and B). In addition to the deformation of the wing shape, extra vein tissues were present in these wings (Supplementary Figure S1B, arrow). Such phenotypes were rescued by co-expressing a *UAS-dNKAP* transgene, confirming the specificity of *dNKAP* knockdown (Supplementary Figure S1C and D). Quantitative polymerase chain reaction (qPCR) analysis further confirmed a reduction in *dNKAP* transcript levels in *dNKAP*-knockdown tissues (Supplementary Figure S1E), and co-expression of *UAS-dNKAP* suppressed this effect (Supplementary Figure S1E). Interestingly, the third-instar larval wing imaginal discs exhibited tissue overgrowth caused by *dNKAP* knockdown (Figure 1A–B’). Measurement of the green fluorescent protein (GFP)-expressing domain in control or *dNKAP*-knockdown discs revealed a significant increase in tissue size caused by *dNKAP* knockdown (Figure 1C). A similar overgrowth phenotype was observed when *dNKAP* was knocked down with the *apterous* (*ap*) Gal4 driver (*ap-Gal4*), which is expressed in the dorsal compartment of the wing disc (Supplementary Figure S2A–B’, quantified in C).

Under normal conditions, *Drosophila* wing imaginal discs maintain a characteristic monolayered architecture. However, most *dNKAP*-knockdown wing discs were disorganized and showed a loss of tissue architecture, consistent with tumorigenic growth (Supplementary Figure S2D–E’). To further evaluate the effect of *dNKAP* knockdown on wing discs, we systematically examined potential changes in cell invasion, cell polarity, cell–cell adhesion, and cytoskeleton organization in *dNKAP*-knockdown tissues. For these analyses, an additional wing Gal4 driver, *en-Gal4*, was used to knock down *dNKAP* expression in the wing posterior compartment, while the expression in the wing anterior compartment can serve as an internal control. The size of third-instar wing imaginal discs was increased upon *dNKAP* knockdown with *en-Gal4* (Supplementary Figure S3). In these *dNKAP*-knockdown wing discs, the anterior–posterior boundary was not as straight as that in wild-type wing discs (Supplementary Figure S3A–B’). Close examination of the wing disc showed that some *dNKAP*-deficient cells from the posterior part migrated to the anterior part (Figure 1D–E’, see arrows in E). Two polarity markers, aPKC and Dlg, were mislocalized in *dNKAP*-deficient cells, suggesting the loss of cell polarity (Figure 1F, arrows). Some *dNKAP*-knockdown cells were also extruded from the basal surface of the epithelium, resulting in the formation of multilayered epithelia (Figure 1F, see DAPI staining). The expression of E-cadherin and armadillo (Arm), two adherens junction markers, was slightly decreased in *dNKAP*-knockdown wing discs (Figure 1G–J’). Accumulation of F-actin (phalloidin) was observed in *dNKAP*-knockdown wing discs (Figure 1K–L’).

Together, these data demonstrate that knockdown of *dNKAP* disrupts the integrity of wing discs and causes tissue overgrowth with several features that are characteristics of tumorigenic growth. They suggest that dNKAP exhibits a tumor-suppressive role in *Drosophila* wing epithelia.

### dNKAP is a nuclear protein and functions to prevent genome instability

**Figure 2:**
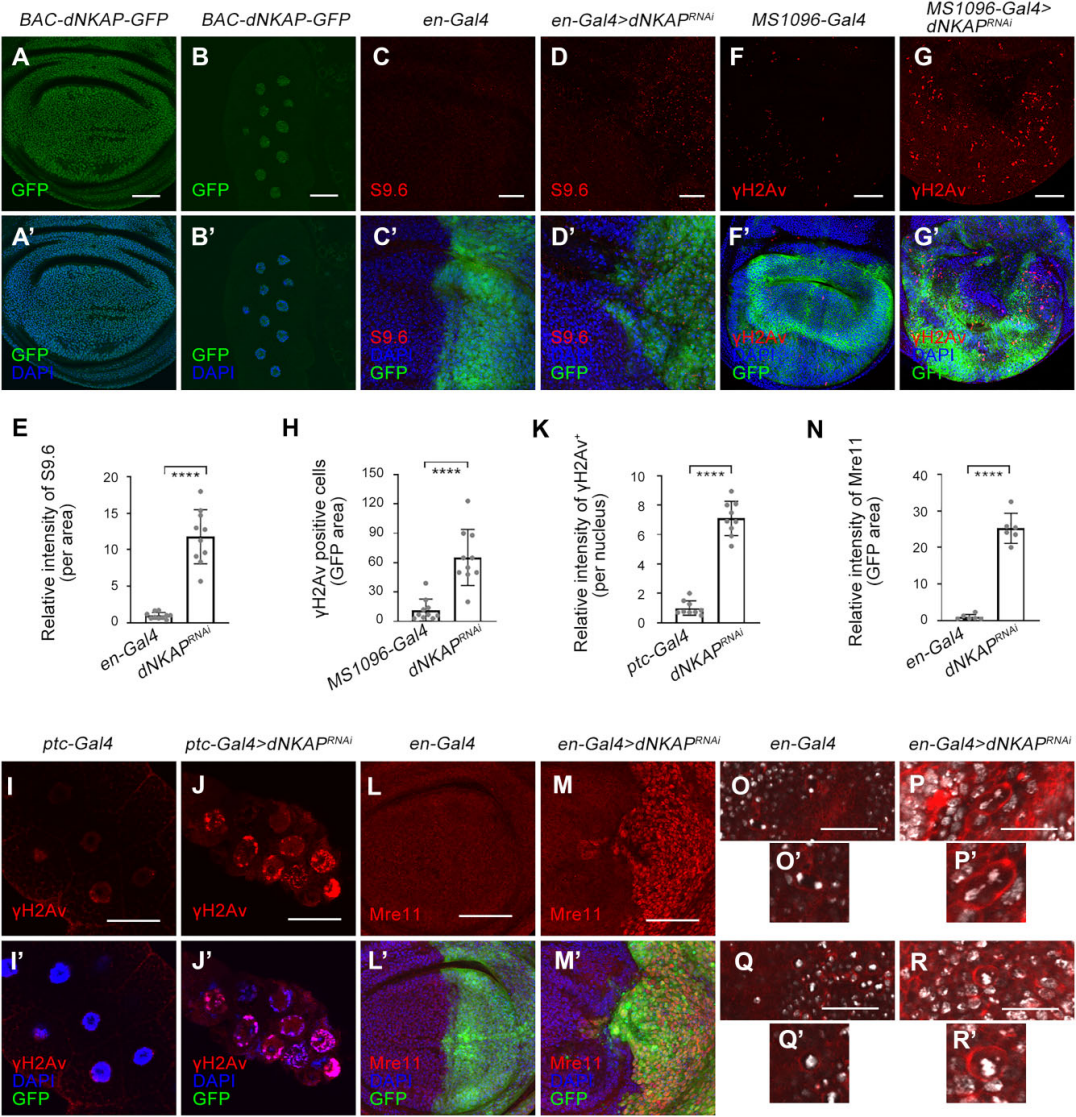
dNKAP localizes to the nucleus and functions to maintain genome stability. (**A**–**B’**) Nuclear localization of dNKAP in mitotic wing disc (**A** and **A’**) and polyploid salivary gland (**B** and **B’**) cells. Scale bar, 50 μm. (**C**–**D’**) Accumulation of R-loops in *dNKAP*-deficient wing disc cells. Scale bar, 20 μm. (**E**) Quantification of S9.6 signals from **C** and **D**. *n = *10. (**F**–**G’**) Increased γH2Av signal in *dNKAP*-deficient wing disc cells. Scale bar, 50 μm. (**H**) Quantification of γH2Av signals from **F** and **G**. *n = *10. (**I**–**J’**) Increased γH2Av signal in *dNKAP*-deficient salivary gland cells. Scale bar, 50 μm. (**K**) Quantification of γH2Av signals from **I** and **J**. *n = *9. (**L**–**M’**) Increased Mre11 protein level in *dNKAP*-deficient wing disc cells. Scale bar, 50 μm. (**N**) Quantification of Mre11 signals from **L** and **M**. *n = *6. Note that three different regions with the same size were calculated per disc. (**O**–**R’**) Chromosome abnormalities in *dNKAP*-deficient wing disc cells. Note the chromosome bridge and micronucleus phenotype in *dNKAP*-deficient cells. Scale bar, 150 μm. GFP was used to mark the knockdown domain. Panels **C**–**D’** and **I**–**J’** show single confocal sections of third-instar wing imaginal discs with posterior side to the right and dorsal side up. Panels **F** and **G** show the maximum projections across image slices. ****P < 0.0001.

NKAP family proteins normally localize in the nucleus and function in multiple processes, including RNA splicing and processing (Burgute et al., 2014). To determine whether dNKAP is also a nuclear protein, we examined the cellular localization of dNKAP using a *BAC-dNKAP-GFP* transgene. In this transgene, *GFP* was fused to *dNKAP* open reading frame, and its expression was under the control of the endogenous *dNKAP* promoter. dNKAP-GFP was found to be primarily located in the nucleus of wing imaginal disc cells (Figure 2A and A’). RNAi of *dNKAP* by *en-Gal4* dramatically reduced the expression of *BAC-dNKAP-GFP* in the wing posterior, confirming the specificity of the GFP signal (Supplementary Figure S4). In addition, dNKAP was also located in the nucleus of non-mitotic polyploid cells, such as salivary gland cells (Figure 2B and B’).

Previous studies showed that deficiency in many mRNP biogenesis factors causes genome instability (Paulsen et al., 2009; Chan et al., 2014; Hamperl and Cimprich, 2014; Naro et al., 2015). This is partly attributed to an increased accumulation of R-loops during transcription (Chan et al., 2014; Hamperl and Cimprich, 2014). We have recently found that human NKAP functions to prevent R-loop-associated genome instability (Zhang et al., 2023). To determine whether depletion of *dNKAP* could result in R-loop accumulation and genome instability, we used an R-loop-specific monoclonal antibody, S9.6, for immunostaining. This antibody is specific for recognizing DNA–RNA hybrids. Our results revealed that RNAi of *dNKAP* by *en-Gal4* triggered a significant accumulation of R-loops in wing imaginal disc cells (Figure 2C–D’ and quantified in E).

R-loop accumulation can lead to DNA damage, which represents one major source of genome instability (Aguilera and Garcia-Muse, 2012; Skourti-Stathaki and Proudfoot, 2014). To monitor whether DNA damage took place due to *dNKAP* knockdown, we used a marker of DNA double-strand breaks, Ser139 phosphorylation of the histone variant H2Av (γH2Av). An increase in nuclear γH2Av levels was observed in disc cells depleted of *dNKAP* (Figure 2F–G’ and quantified in H). Moreover, knockdown of *dNKAP* in the salivary gland using *ptc-Gal4* also led to an increased level of γH2Av (Figure 2I–J’ and quantified in K). This confirmed that depletion of *dNKAP* caused DNA damage. Higher expression levels of DNA damage-responsive genes are often observed when DNA lesions occur (Saini et al., 2012). We then examined the expression levels of two DNA damage-responsive genes, *Mre11* and *Nbs*, in *dNKAP*-deficient wing disc cells. Both *Mre11* and *Nbs* were significantly upregulated in *dNKAP*-knockdown cells, indicating that *dNKAP* depletion indeed results in DNA lesions and induces DNA damage response (Figure 2L–M’ and quantified in N; Supplementary Figure S5).

Increased DNA damage is associated with chromosome abnormalities. We found that a few *dNKAP*-deficient cells showed chromosome segregation defects, including anaphase chromatin bridges (Figure 2O–P’, found in 11 discs from a total of 34 discs). Wing disc cells containing a micronucleus in addition to a larger nucleus were also detected upon *dNKAP* knockdown (Figure 2Q–R’, found in 4 discs from a total of 34 discs).

Taken together, these results illuminate that dNKAP acts to maintain genome integrity and suggest that loss of this function might cause tumorigenic growth.

### dNKAP knockdown induces JNK-dependent apoptosis

**Figure 3:**
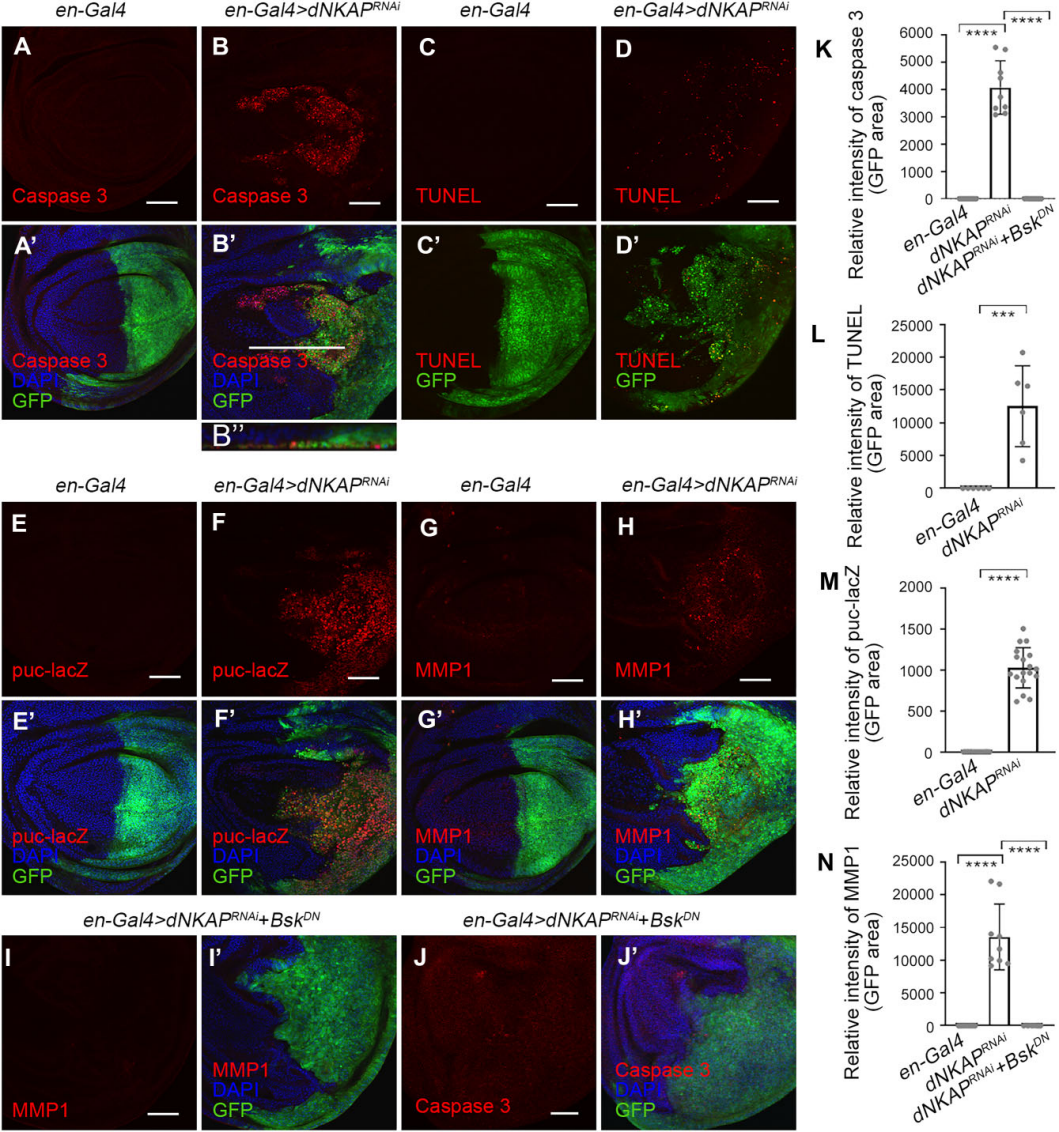
*dNKAP* depletion induces JNK-dependent apoptosis. (**A**–**B’’**) Increased caspase 3 staining in *dNKAP*-knockdown wing discs. (**C**–**D’**) Increased TUNEL staining in *dNKAP*-knockdown wing discs. (**E**–**F’**) Upregulation of *puc-lacZ* in *dNKAP*-knockdown wing discs. (**G**–**H’**) Upregulation of MMP1 in *dNKAP*-knockdown wing discs. (**I**–**J’**) Reducing JNK activity suppresses ectopic MMP1 expression and apoptosis in *dNKAP*-knockdown wing discs. (**K**) Quantification of caspase 3 signals from **A, B**, and **J**. *n = *9. Note that three different regions with the same size were calculated per disc. (**L**) Quantification of TUNEL signals from **C** and **D**. *n = *6. Note that three different regions with the same size were calculated per disc. (**M**) Quantification of puc-lacZ signals from **E** and **F**. *n = *18. Note that three different regions with the same size were calculated per disc. (**N**) Quantification of MMP1 signals from **G, H**, and **I**. *n = *9. Note that three different regions with the same size were calculated per disc. GFP was used to mark the knockdown domain. Panels **A**–**B’** and **C**–**J’** show single confocal sections of third-instar wing imaginal discs with posterior side to the right and dorsal side up. **B’’** shows the optical cross-section of the wing imaginal disc with apical side up. Scale bar, 50 μm. ***P < 0.001, ****P < 0.0001.

DNA damage and genome instability can inherently have adverse effects on cells, and such effects often lead to apoptosis (Tubbs and Nussenzweig, 2017). To determine whether *dNKAP* knockdown induces apoptosis in wing imaginal discs, we utilized an anti-cleaved caspase 3 antibody to detect apoptotic cells. RNAi of *dNKAP* by *en-Gal4* resulted in high levels of cleaved caspase 3 staining in cells of the posterior compartment of wing discs (Figure 3A–B’ and quantified in K). Most of these apoptotic cells were located on the basal side of the wing disc (Figure 3B’’). In addition, we noticed that some apoptotic cells were present outside of the *en-Gal4*-expressing domain (Figure 3B and B’). We further confirmed that apoptotic cells were present in *dNKAP*-knockdown tissues by TUNEL assay (Figure 3C–D’ and quantified in L).

The c-Jun N-terminal kinase (JNK) signaling pathway is involved in the induction of apoptosis in response to DNA damage and other stresses (Weston and Davis, 2007). To examine the role of the JNK pathway in *dNKAP* depletion-mediated apoptosis, we first monitored JNK activity by analyzing the expression level of the *puc-lacZ* reporter gene in *dNKAP*-knockdown and control tissues. A clear upregulation of *puc-lacZ* expression was observed in the posterior compartment of the wing disc when *dNKAP* was depleted by RNAi with *en-Gal4*, suggesting activation of the JNK pathway in *dNKAP*-depleted cells (Figure 3E–F’ and quantified in M). Similarly, MMP1, another JNK signaling reporter, was upregulated in *dNKAP*-depleted tissues (Figure 3G–H’ and quantified in N). To further investigate whether *dNKAP* depletion-caused apoptosis was dependent on activation of the JNK pathway, we reduced JNK activity by expressing a dominant-negative form of Bsk in *dNKAP*-depleted tissues. As expected, MMP1 protein levels in *dNKAP*-deficient cells decreased significantly when JNK was inhibited (Figure 3I and I’ and quantified in N). Co-expression of *UAS-Bsk^DN^* was able to largely rescue the apoptotic cell death phenotype caused by *dNKAP* depletion in the wing disc (Figure 3J and J’ and quantified in K).

Moreover, we generated a loss-of-function mutant allele, *dNAKP^1^*, by using homologous recombination (Supplementary Figure S6A). This mutant is a functional null allele, as the entire coding region of *dNAKP* was deleted and replaced with a *mini-white* gene (Supplementary Figure S6A). The genomic deletion was verified by PCR using two pairs of primers (Supplementary Figure S6B). Consistently, reverse transcription–PCR analyses confirmed the absence of *dNKAP* transcripts in *dNKAP^1^* homozygous mutants (Supplementary Figure S6C). *dNKAP^1^* mutants were homozygous lethal. Detailed analyses revealed that homozygous *dNKAP^1^* mutants died at late embryonic/first-instar larval stages. Expression of *UAS-dNKAP* with a ubiquitously expressed *Tubulin-Gal4* (*Tub-Gal4*) was capable of fully rescuing the homozygous lethality of *dNAKP^1^* mutants, indicating that the lethality was due to the specific loss of *dNKAP* (Supplementary Table S1). We then made use of the FLP/FRT system and generated mitotic clones of homozygous *dNKAP^1^* mutant cells in wing imaginal discs during larval development. Homozygous mutant clones and their twin spots were marked by the absence of GFP and the presence of two copies of GFP, respectively. Surrounding tissues were labeled with one copy of GFP. Anti-cleaved caspase 3 antibody was used to label the apoptotic cells. When *dNKAP^1^* mutant clones were induced during the first-instar larval stage (34–36 h after egg laying) and analyzed in wandering third-instar larvae (122–124 h after egg laying), we failed to recover any *dNKAP* homozygous mutant clone (Supplementary Figure S6E–E’’’). Large clones were observed for the wild-type control under the same conditions (Supplementary Figure S6D–D’’’). When we shortened the time between clone induction and analysis by inducing clones later at 70–72 h after egg laying, we were still unable to observe *dNKAP^1^* homozygous mutant cells (Supplementary Figure S6F–F’’’ and G–G’’’). However, in optical cross sections through the wing discs, it was apparent that a few cells stained positive for cleaved caspase 3 accumulated at the basal side of the wing disc under both conditions (Supplementary Figure S6D’’’’, D’’’’’, E’’’’, E’’’’’, F’’’’, F’’’’’, G’’’’, and G’’’’’). This indicates that *dNKAP^1^* mutant cells are likely eliminated and extruded to the basal surfaces of the wing disc. The elimination of *dNKAP* mutant clones in the wing disc indicates that these mutant clones are defective in cell survival in a wild-type background. We next used the ‘Minute technique’ to recover *dNKAP* mutant clones. This technique gave *dNKAP* mutant clones a growth advantage over their neighbors. With this method, wild-type clones grew to large sizes (Supplementary Figure S7A–A’’’). In contrast, only small patches of *dNKAP* mutant cells were observed over the same time period (Supplementary Figure S7B–B’’’). These *dNKAP* mutant cells also accumulated at the basal surface of the epithelium and had abnormal pyknotic nuclei, a typical feature of wing disc cells undergoing apoptosis (Supplementary Figure S7B’’’’). Consistent with this, extensive caspase 3 staining was observed in these *dNKAP* mutant clone cells located on the basal side of the wing disc (Supplementary Figure S7B’’’’).

Altogether, these results indicate that *dNKAP* knockdown induces JNK-dependent apoptosis in the wing disc. In addition, *dNKAP* is an essential gene, as complete loss-of-function mutation of *dNKAP* causes early lethality during development.

### dNKAP knockdown causes aberrant cell proliferation

**Figure 4:**
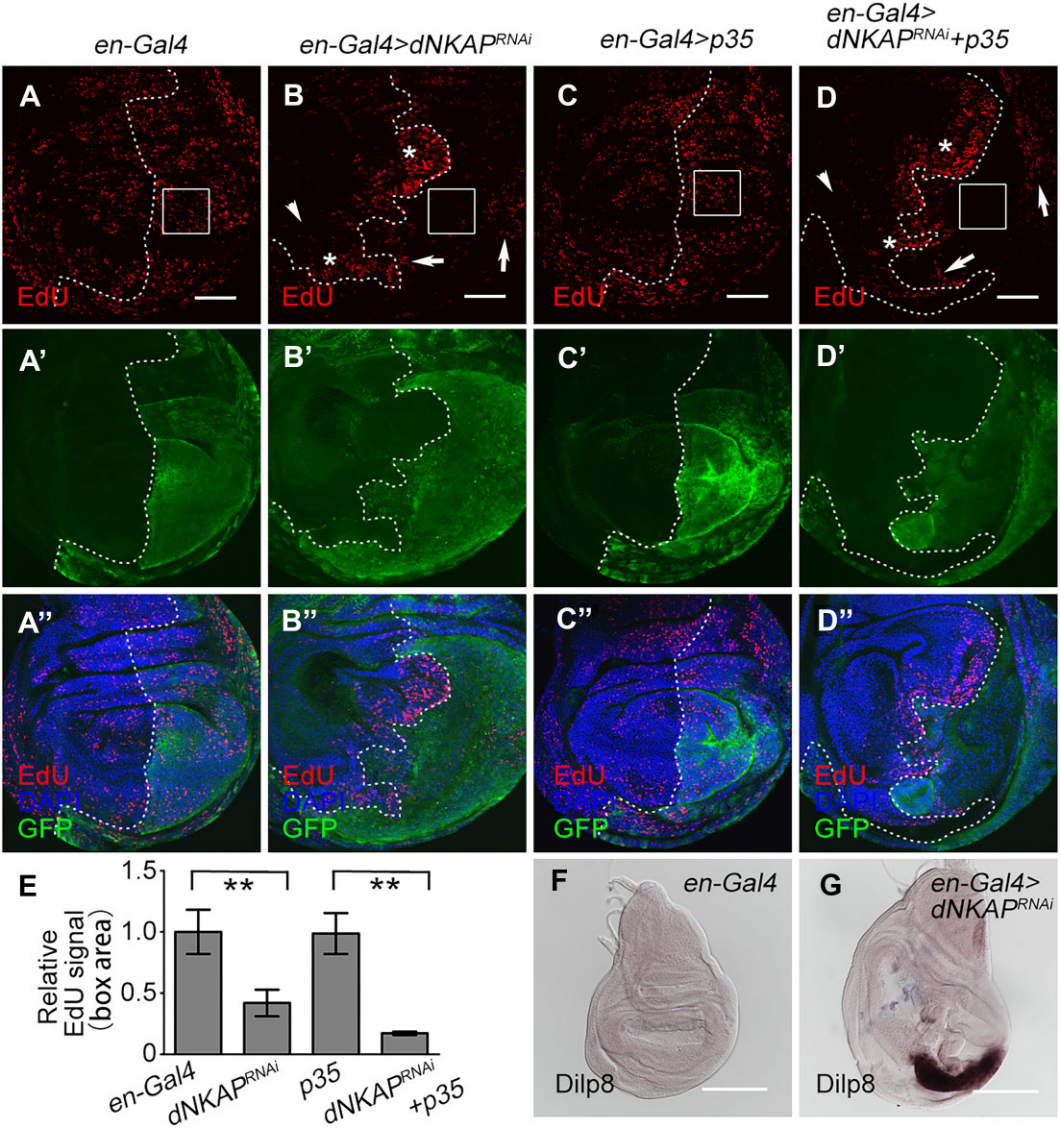
Aberrant cell proliferation in *dNKAP*-knockdown wing discs. (**A**–**D’’**) Decreased EdU signal in a population of *dNKAP*-depleted cells. Boxes indicate the pouch region. Note that EdU signals are increased in a patch of *dNKAP*-depleted cells (indicated by arrows) and the adjacent wild-type cells (indicated by stars) along the anterior–posterior boundary but decreased in the wild-type cells far from *dNKAP*-depleted cells in the anterior compartment (indicated by arrowheads). GFP was used to mark the knockdown domain. Shown are single confocal sections of third-instar wing imaginal discs with posterior side to the right and dorsal side up. Scale bar, 50 μm. (**E**) Quantification of EdU signals in the boxed area of the wing disc. *n = *3. **P < 0.01. (**F** and **G**) Increased *Dilp8* mRNA level in *dNKAP*-knockdown wing discs. Scale bar, 100 μm.

Next, we investigated cell proliferation in *dNKAP*-knockdown discs. Analysis of EdU incorporation revealed that cell proliferation appears in a heterogeneous manner (Figure 4A–D’’). The number of S-phase cells in the wing pouch area was reduced upon *dNKAP* knockdown in the *en-Gal4*-expressing domain compared with the control, suggesting that *dNKAP* knockdown caused a slower cell cycle progression (Figure 4A–B’’, EdU signals in the boxed area quantified in E). Similar results were obtained when apoptosis was blocked by overexpressing p35 (Figure 4C–D’’ and quantified in E). In addition, increased EdU signals were observed in a patch of *dNKAP*-depleted cells in the hinge region (Figure 4B and D, arrows). Interestingly, the adjacent wild-type cells along the anterior–posterior boundary showed higher EdU incorporation, indicating that knockdown of *dNKAP* causes non-autonomous cell overproliferation (Figure 4B and D, stars). However, anterior wild-type cells far from the *dNKAP*-knockdown domain proliferated slowly (Figure 4B and D, arrowdeads). It appears that the depletion of *dNKAP* in the posterior wing disc generates a non-autonomous effect to coordinate tissue growth among different cell populations, a previously reported stress-responsive phenomenon. To further confirm these effects, we performed EdU incorporation experiments in *MS1096-Gal4*-driven *dNKAP*-knockdown wing discs in the presence or absence of p35. Consistently, cell proliferation occurred in a heterogeneous manner (Supplementary Figure S8A–D’’), including reduced EdU signals in *dNKAP*-depleted cells within the wing pouch area (Supplementary Figure S8B’’ and D’’, boxes), increased EdU signals in a patch of *dNKAP*-depleted cells within the hinge region (Supplementary Figure S8B’’ and D’’, arrows), and a few adjacent wild-type cells (Supplementary Figure S8B’’ and D’’, stars).

As described above, the *dNKAP*-knockdown larvae had delayed puparium formation, which raised the possibility that these *dNKAP*-deficient cells were able to grow and divide continuously beyond the normal proliferation stages. It has been shown that the developmental delay caused by damaged wing discs is mediated by Dilp8, which is an insulin/insulin-like growth peptide hormone produced and released by damaged wing discs (Colombani et al., 2012; Garelli et al., 2012; Katsuyama et al., 2015). We then used RNA *in situ* hybridization to examine the expression of *Dilp8*. Strong ectopic expression of *Dilp8* RNA was present in the *dNKAP*-depleted region (Figure 4F and G). Further RNA fluorescence *in situ* hybridization analysis confirmed that Dilp8 was indeed induced upon *dNKAP* knockdown in the posterior compartment of the wing disc, which was marked by the GFP signal (Supplementary Figure S9A–B’’’). Moreover, we tested whether induction of Dilp8 mediated the developmental delay in *dNKAP*-knockdown larvae. Co-depletion of *dNKAP* and *Dilp8* partially rescued the developmental delay caused by *dNKAP* depletion (Supplementary Figure S9C). These results suggest that these slow-cycling *dNKAP*-deficient cells can proliferate for a longer time, even at a slower rate.

### The Notch and JAK/STAT signaling pathways are required for dNKAP knockdown-induced tumorigenic growth

**Figure 5:**
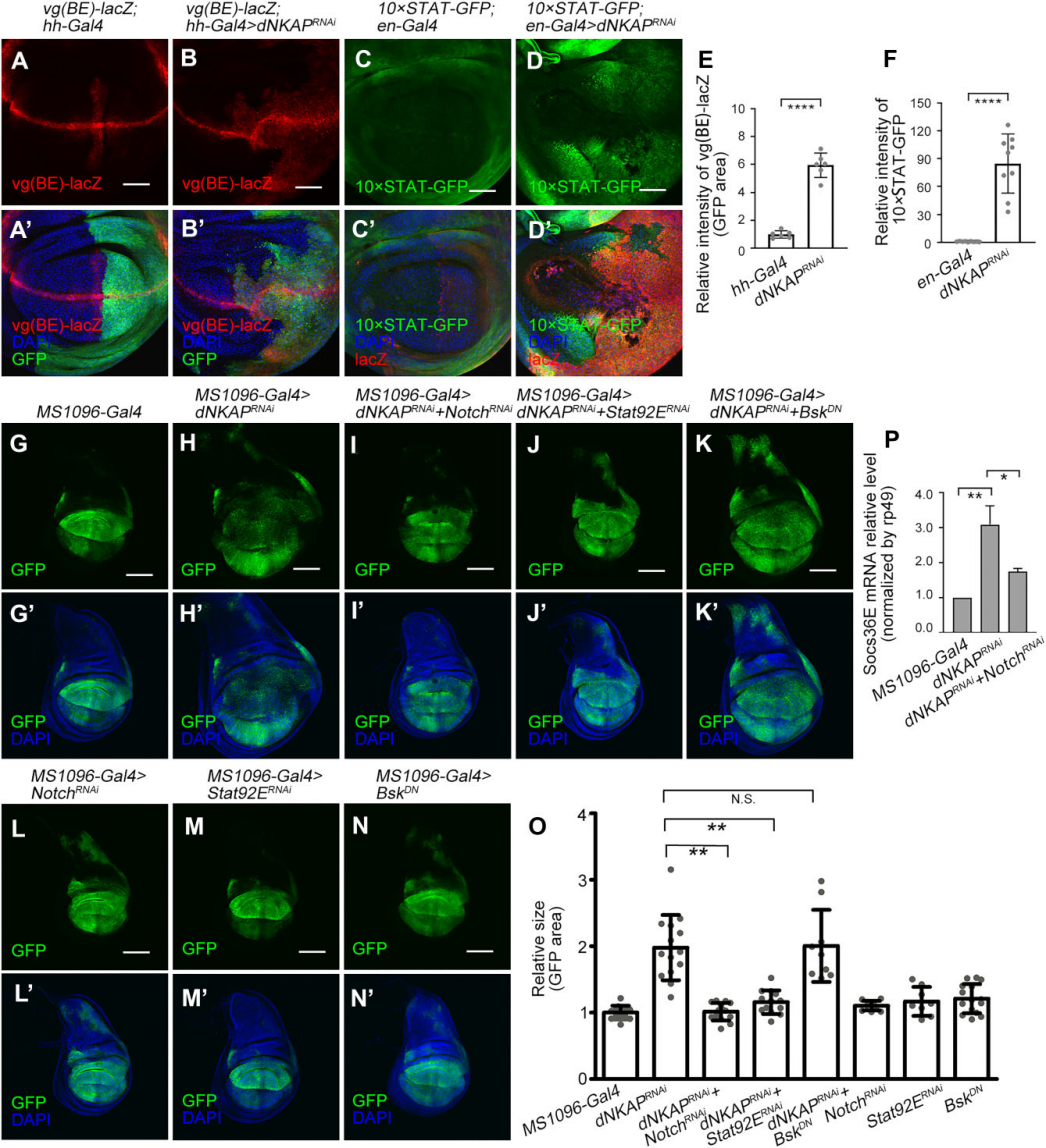
Notch and JAK/STAT signaling pathways contribute to the tumorigenic growth of *dNKAP*-knockdown tissues. (**A**–**D’**) Upregulation of *vg(BE)-lacZ* (**A**–**B’**) and *10×STAT-GFP* (**C**–**D’**) in *dNKAP*-knockdown wing discs. Shown are single confocal sections of third-instar wing imaginal discs with posterior side to the right and dorsal side up. Scale bar, 50 μm. (**E**) Quantification of *vg(BE)*-*lacZ* signals from **A** and **B**. *n = *6. Note that three different regions with the same size were calculated per disc. (**F**) Quantification of *10×STAT-GFP* signals from **C** and **D**. *n = *9. Note that three different regions with the same size were calculated per disc. (**G**–**N’**) Reducing Notch or JAK/STAT signaling, but not JNK signaling, suppresses tumorigenic growth of *dNKAP*-knockdown wing discs. Shown are single confocal sections of third instar wing imaginal discs with dorsal side up. Scale bar, 100 μm. (**O**) Quantification of GFP-positive area in wing discs with the indicated genotypes. *n = *12, 14, 14, 13, 9, 10, 8, and 15 for each genotype, respectively. The value was calculated by normalizing to the mean area of the control discs. (**P**) Knockdown of *Notch* suppresses the upregulated Socs36E level in *dNKAP*-depleted wing discs. Quantification of Socs36E levels in wing discs with the indicated genotypes. *n = *3. GFP or lacZ was used to mark the knockdown domain. N.S. indicates no significance. *P < 0.05, **P < 0.01, ****P < 0.0001.

Multiple conserved signaling pathways, including the JNK, Notch, and Janus kinase/signal transducer and activator of transcription (JAK/STAT) pathways, have been implicated in growth control and tumorigenesis in *Drosophila* wing epithelia (Herranz and Cohen, 2017; Tamori and Deng, 2017; Enomoto et al., 2018; Richardson and Portela, 2018). As shown previously, the JNK signaling pathway was activated in *dNKAP*-deficient cells (Figure 3E–H’). Notch signaling was strongly upregulated, as monitored by the increased expression of its reporters, *vg(BE)-lacZ* and *Su(H)-lacZ* (Figure 5A–B’ and quantified in E; Supplementary Figure S10A–B’ and quantified in E). Furthermore, the expression of *10×STAT-GFP*, a reporter of JAK/STAT signaling, was also highly upregulated in *dNKAP*-knockdown cells (Figure 5C–D’ and quantified in F). Consistently, *upd-lacZ*, another JAK/STAT reporter, was expressed at a higher level in *dNKAP*-knockdown cells (Supplementary Figure S10C–D’ and quantified in F). Based on these results, we conclude that multiple growth-related signaling pathways are activated in the absence of *dNKAP*.

We then examined whether the Notch, JAK/STAT, and JNK signaling pathways contribute to the tumorigenic growth of *dNKAP*-depleted discs. Knockdown of *Notch* or *Stat92E* by RNAi suppressed the tissue overgrowth phenotype in *dNKAP*-knockdown wing discs (Figure 5G–J’ and quantified in O). RNAi of *Notch* or *Stat92E* by *MS1096-Gal4* did not dramatically affect tissue size (Figure 5L–M’ and quantified in O). RNAi of *Notch* or *Stat92E* by *en-Gal4* led to the reduced levels of Notch protein and *10×STAT-GFP* reporter, respectively, confirming the specificity of both RNAi lines (Supplementary Figure S11A–B’’’). Furthermore, we performed qPCR analysis to examine whether knockdown of Notch affects the expression level of Socs36E, a JAK/STAT target, in *dNKAP*-depleted wing discs. Compared with *dNKAP* depletion alone, co-depletion of *dNKAP* and *Notch* was able to suppress the upregulation of *Socs36E* expression (Figure 5P), suggesting that Notch signaling might function upstream of JAK/STAT signaling in regulating the *dNKAP*-knockdown phenotype. Reducing JNK activity by overexpressing *Bsk^DN^* did not suppress the tumorigenic growth of *dNKAP*-knockdown wing discs (Figure 5K, K’, N, and N’). However, cell-invasive behavior in *dNKAP*-knockdown discs was completely suppressed when *Bsk^DN^* was co-expressed (Supplementary Figure S12A–D’, arrows indicating invasive cells). This inhibition was not due to the block of cell death, as prevention of apoptosis by co-expressing p35 did not change cell-invasive behavior in *dNKAP*-knockdown wing discs (Supplementary Figure S12E–F’, arrows indicating invasive cells).

In summary, these results demonstrate that the Notch and JAK/STAT signaling pathways contribute to the tumorigenic growth of *dNKAP*-knockdown tissues.

### Transcriptional profiling confirms the activation of multiple signaling pathways and reveals metabolic changes in dNKAP-knockdown wing discs

**Figure 6:**
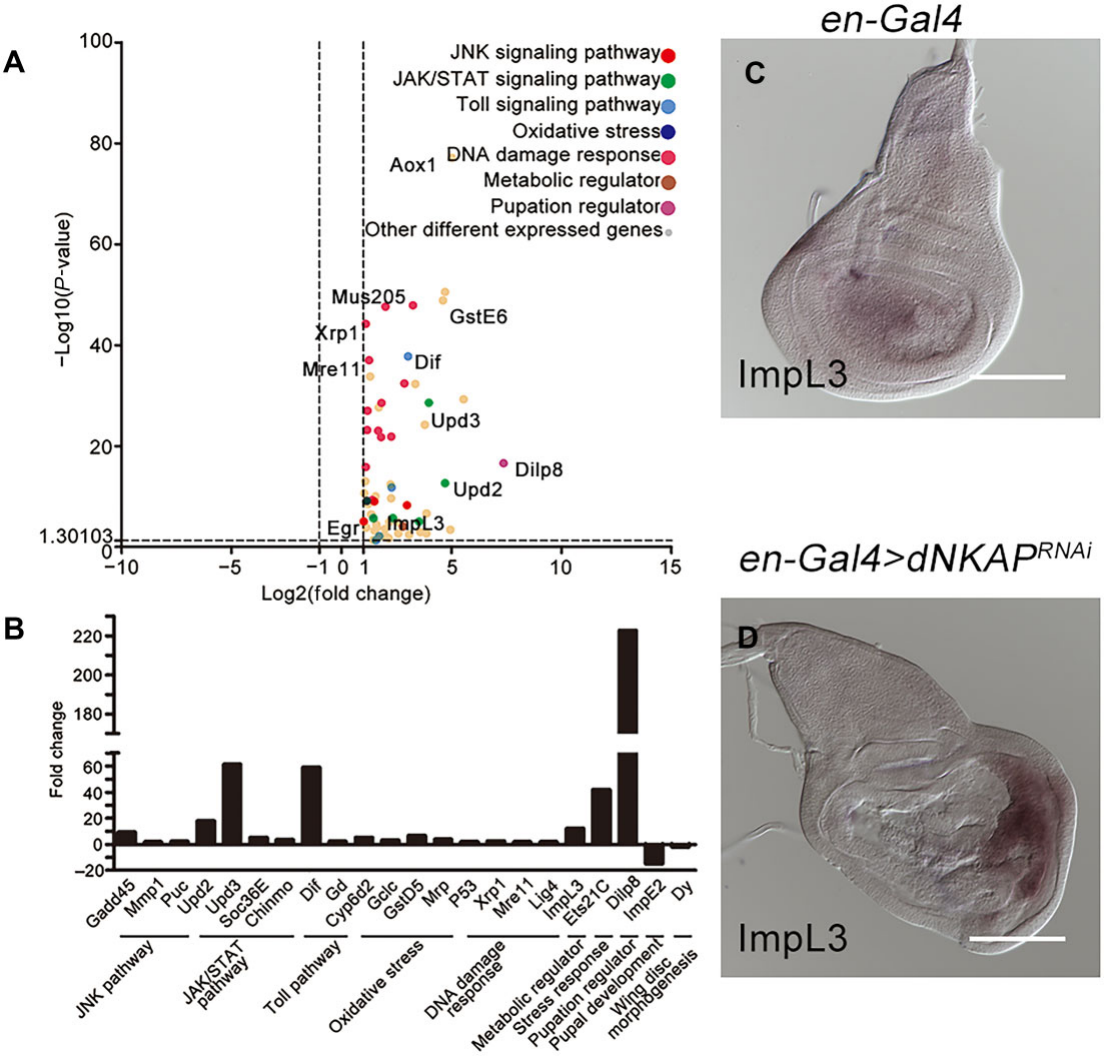
Transcriptome analysis of *dNKAP*-knockdown tissues. (**A**) RNA-Seq dataset of differentially expressed genes in *dNKAP*-knockdown wing discs compared to the control discs. Pathways activated in *dNKAP*-knockdown tissues are highlighted. (**B**) qPCR analysis of selected differentially expressed genes in *dNKAP*-knockdown tissues. Fold changes represent the expression levels in *dNKAP*-knockdown wing discs relative to the control discs. Data shown are the average values from three technical replicates. (**C** and **D**) Increased expression of *ImpL3* in *dNKAP*-knockdown wing discs. Scale bar, 100 μm.

To determine the global effects of dNKAP on gene expression and gain more molecular insights into the tumorigenic growth phenotype caused by *dNKAP* knockdown in the wing disc, we carried out a genome-wide RNA-sequencing (RNA-Seq) analysis. RNAs isolated from three biological replicas of both *MS1096-Gal4*-driven *dNAKP*-knockdown and control wing discs were subjected to RNA-Seq analysis. This analysis revealed that 433 genes were misregulated at least twofold upon RNAi of *dNKAP*, of which 362 were upregulated and 71 were downregulated (Supplementary Table S2). These differentially expressed genes were further classified according to their identity and function. Gene Ontology analysis showed that the differentially expressed gene list was highly enriched for genes associated with oxidoreductase activity, response to stress, small molecule metabolic process, sulfur compound metabolic process, transferase activity (transferring alkyl or aryl), and extracellular region (Supplementary Table S3). Through manual analysis, we found that *dNKAP*-knockdown wing disc cells had increased expression of components of our previously characterized signaling pathways, including the JNK, JAK/STAT, and DNA damage response pathways (Figure 6A). Upregulation of *Dilp8* transcript levels was evident in the RNA-Seq results, while increased expression of Notch signaling components was not detected (Figure 6A). Interestingly, many components of the Toll pathway and the oxidative stress response pathway, including the NF-kappaB transcription factor Dif, displayed increased expression upon *dNKAP* knockdown (Figure 6A). As a metabolic regulator, lactate dehydrogenase (LDH) is often upregulated in tumor cells to promote glycolysis. ImpL3, the *Drosophila* homolog of LDH, was also expressed at a higher level in *dNKAP*-depleted tissues, indicating that loss of *dNKAP* drives metabolic rewiring (Figure 6A). Thus, *dNKAP* depletion appears to affect the expression and activity of the components of multiple signaling pathways as well as cellular metabolism in the developing wing.

Our RNA-Seq results were experimentally validated by qPCR analysis of selected genes representative of various pathways. Their mRNA levels measured by qPCR showed close agreement with RNA-Seq data (Figure 6B). In addition, RNA *in situ* hybridization analysis also confirmed that ImpL3 expression was specifically increased in *dNKAP*-depleted tissues (Figure 6C and D). To further examine the effect of ImpL3 induction on tissue overgrowth caused by *dNKAP* knockdown, we co-depleted *ImpL3* and *dNKAP* in wing discs using *MS1096-Gal4* and examined wing disc size. Our results showed that knockdown of *ImpL3* failed to rescue the overgrowth phenotype in *dNKAP*-depleted wing discs, indicating the presence of other crucial metabolic factors for tumorigenic growth (Supplementary Figure S13A–D’ and quantified in E). Together, our transcriptional profile identified many differentially expressed genes in the wing disc when *dNKAP* was knocked down and revealed a global gene expression pattern of *dNKAP*-knockdown tissues.

NKAP interacts with multiple components of the spliceosome and has a role in regulating mRNA splicing (Burgute et al., 2014). We next examined how *dNKAP* loss affected mRNA splicing in wing discs. For this, we used the above RNA-Seq datasets to perform mRNA splicing analysis. From this analysis, a total of 177 alternative splicing events with significant changes were observed between *dNKAP*-knockdown and control discs, with intron retention being the most frequently altered splicing event followed by exon skipping, as well as other splicing defects, including alternative 5′-splice site usage, alternative 3′-splice site usage, and changes in mutually exclusive exon usage (Supplementary Figure S14A and Table S5). Further Kyoto Encyclopedia of Genes and Genomes (KEGG) analysis revealed that the top-enriched pathways in the alternatively spliced gene list were ABC transporters, citrate cycle, pentose phosphate pathway, and glycosphingolipid biosynthesis-ganglio series (Supplementary Figure S14B). Through manual analysis, we also examined whether the key genes analyzed in the above experiments were altered in the splicing of their mRNA transcripts. These genes include *Dlg, aPKC, Arm, E-cadherin, Mre11, Nbs, puc, MMP1, Dilp8, vg, Socs36E, ImpL3, Dif, p53, Xrp1, Ets21C, ImpE2*, and *Dy*. Interestingly, *Xrp1* showed significant changes in mRNA splicing (Supplementary Table S5). These results suggest that dNKAP can regulate mRNA splicing and influence gene expression at multiple levels.

### Synergies between dNKAP knockdown and oncogenic form of Ras, Notch, or Yki in inducing tumorigenic growth

**Figure 7:**
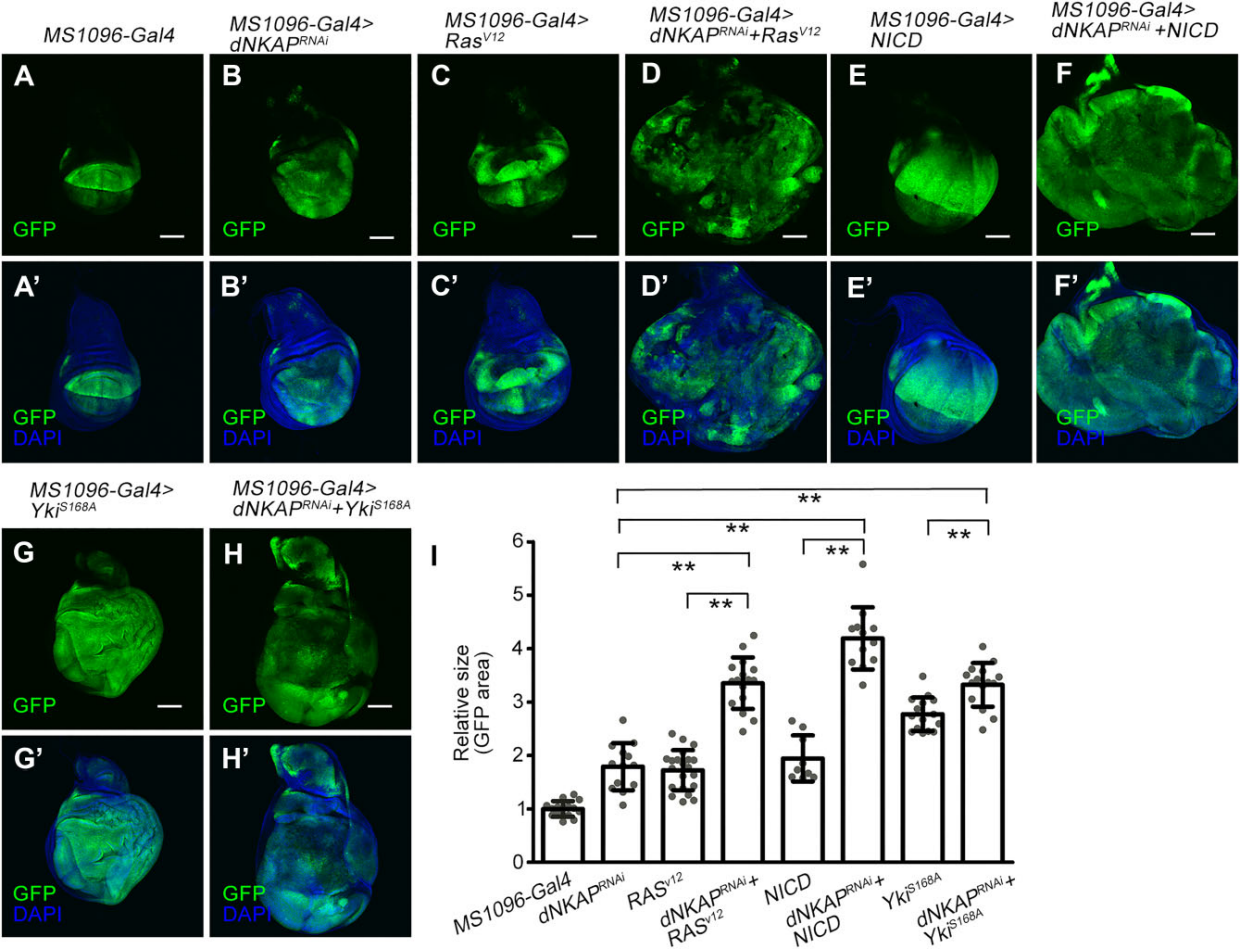
Synergies between *dNKAP* depletion and oncogenic form of Ras, Notch, or Yki in promoting tumorigenic growth. (**A**–**H’**) Ectopic expression of *Ras^V12^, NICD*, or *Yki^S168A^* in *dNKAP*-knockdown tissues induces massive tissue overgrowth. GFP was used to mark the knockdown domain. Shown are single confocal sections of third-instar wing imaginal discs with dorsal side up. Scale bar, 100 μm. (**I**) Quantification of GFP-positive area in wing discs with the indicated genotypes. *n = *15, 13, 21, 16, 9, 12, 15, and 15 for each genotype, respectively. The value was calculated by normalizing to the mean area of the control disc. **P < 0.01.

Oncogenic gene interactions have been extensively studied in both *Drosophila* wing and eye epithelial tissues (Enomoto et al., 2018; Richardson and Portela, 2018). Using these systems, it has been reported that oncogenic forms of Ras, Notch, or Yki synergize with various tumor suppressor mutations or oncogene overexpression, such as Scrib, Src, Mef2, miR-8, BAP, and PP6 protein complexes, to drive tumorigenesis and invasion (Brumby and Richardson, 2003; Pagliarini and Xu, 2003; Pallavi et al., 2012; Ho et al., 2015; Ma et al., 2017; Song et al., 2017; Xie et al., 2017; Sander et al., 2018). Therefore, we examined whether ectopic expression of oncogenic form of Ras, Notch, or Yki affects the growth of *dNKAP*-knockdown wing discs. As shown above, depletion of *dNKAP* by *MS1096-Gal4* caused a tissue overgrowth phenotype (Figure 7A–B’ and quantified in I). Interestingly, co-expression of *dNKAP* RNAi with *Ras^V12^, NICD*, or *Yki^S168A^* induced massive tissue overgrowth, while expression of *Ras^V12^, NICD*, or *Yki^S168A^* alone did not cause such dramatic tissue overgrowth (Figure 7C–H’ and quantified in I). Furthermore, wing discs expressing *Ras^V12^, NICD*, or *Yki^S168A^* with *dNKAP* RNAi had many extra folds and were more disorganized (Figure 7C–H’). In some discs, GFP-positive cells occupied almost the entire organ, and the surrounding wild-type tissue was outcompeted (Figure 7C–H’). To further explore the role of dNKAP in tumor development, we examined whether *dNKAP* overexpression in wing discs could suppress the tumorigenic growth in an oncogenic Ras activation background. Surprisingly, overexpression of *dNKAP* in wing discs using *MS1096-Gal4* promoted tumorigenic growth caused by *Ras^V12^* expression, indicating that tight regulation of *dNKAP* expression is important in the context of Ras activation (Supplementary Figure S15A–D’ and quantified in E). Taken together, these data indicate that oncogenic Ras, Notch, or Yki enhances tumorigenic growth in *dNKAP*-knockdown wing epithelia.

## Discussion

Our study demonstrated that *Drosophila dNKAP* plays a tumor-suppressive role by preventing genome instability. First, dNKAP functions to prevent tissue overgrowth and maintain epithelial integrity. Second, *dNKAP*-deficient cells show the accumulation of R-loops and DNA damage, thereby losing genome stability. Consistently, apoptotic and slow-cycling cells are present in *dNKAP*-knockdown tissues. Third, the activation of Notch and JAK/STAT signaling is involved in the tumorigenic growth of *dNKAP*-depleted tissues, and the upregulation of the JNK pathway is required for cell-invasive behavior in these tissues. Fourth, transcriptome analysis results show that the activation of multiple signaling pathways and metabolic changes occur in *dNKAP-*knockdown tissues. Finally, the oncogenic form of Ras, Notch, or Yki enhances tumorigenesis in *dNKAP*-knockdown wing epithelia.

*Drosophila dNKAP* is an essential gene, and the complete loss-of-function mutation of *dNKAP* prevents cell survival and causes embryonic/early larval lethality. However, *dNKAP* depletion promotes tumorigenic growth despite the occurrence of cell death, as *dNKAP*-knockdown wing disc cells display characteristics of neoplastic tumors. Genome instability is the driving force for tumorigenesis and is associated with the heterogeneity of cells during tumor initiation (Negrini et al., 2010). We found that depletion of *dNKAP* results in chromosome abnormalities in *Drosophila* wing epithelial cells, although the frequency of cells with abnormal chromosomes and micronuclei is low. Interestingly, *dNKAP*-deficient cells show persistent accumulation of R-loops, which is consistent with our recent findings on human NKAP (Zhang et al., 2023). R-loops can cause genome instability by inducing DNA damage or interfering with DNA replication (Aguilera and Garcia-Muse, 2012; Naro et al., 2015). Staining with the DNA damage marker γH2Av confirms that depletion of dNKAP results in DNA damage in both wing imaginal disc and salivary gland cells. The link between splicing factors and R-loop formation has been well documented in yeast and mammalian cells (Aguilera and Garcia-Muse, 2012). One potential mechanism is that splicing factors function to bind to the nascent RNA transcript to prevent the formation of R-loop structures (Aguilera and Garcia-Muse, 2012; Naro et al., 2015). In mammalian cells, NKAP functions to promote the activation of NF-kappaB signaling. In our RNA-Seq data, the expression level of Dif was upregulated in *dNKAP*-knockdown wing discs compared to the control, indicating that loss of dNKAP activates NF-kappaB signaling in *Drosophila.* This is in contrast to the functions of mammalian NKAP. Thus, the role of dNKAP in regulating NF-kappaB signaling in *Drosophila* might be complicated.

Upon *dNKAP* knockdown, two growth-promoting pathways, the Notch and JAK/STAT pathways, are activated and required for tumorigenic growth. In addition, the JNK pathway induces both apoptosis and cell invasion in *dNKAP*-depleted tissues. The changes in Notch and JAK/STAT pathway activities are likely associated with genome instability and tumorigenesis. It is possible that the pleiotropic effects caused by *dNKAP* depletion are due to genome instability in wing disc cells. The activation of multiple signaling pathways and metabolic changes determined by the transcriptome analysis further support this notion.

*dNKAP*-knockdown tissues share many characteristics of epithelial tumors, including disorganization of the epithelium, cell invasion, upregulation of MMP1, and loss of cell polarity. However, apoptotic cells and slow-cycling cells are also present in *dNKAP*-depleted tissues. We show that knockdown of *dNKAP* causes wing disc overgrowth. At first glance, this seems inconsistent with the reduced cell proliferation and the increased levels of cell death observed in *dNKAP*-knockdown tissues. There are two explanations for this. First, *dNKAP*-knockdown wing disc cells exhibit heterogeneity with distinct behaviors of different cell populations. For example, we showed that a subpopulation of cells in *dNKAP*-knockdown discs have elevated EdU signals, indicating increased cell proliferation. Thus, it is likely that cells with reduced proliferation or undergoing apoptotic cell death could stimulate the proliferation of surrounding cells, even if they are genetically identical. Second, control larvae normally reach the wandering third instar at 120 h after egg laying, but the *dNKAP*-knockdown larvae continue to grow through an extended larval stage. Thus, the discs of *dNKAP*-knockdown larvae could reach a size much larger than control discs. Moreover, the overgrowing capacity of *dNKAP*-knockdown tissues is relatively limited compared to that of previously reported neoplastic or hyperplastic wing discs (Bilder, 2004). This suggests that *dNKAP*-knockdown wing disc cells resemble human tumor cells in the early stage of tumor development to a certain degree (Halazonetis et al., 2008). Previous studies in different stages of human tumors revealed that DNA damage response acts as an anti-cancer barrier to delay or prevent cancer in early tumorigenesis (Halazonetis et al., 2008). It is likely that the enhanced genome instability in these *dNKAP*-deficient cells may enable them to overcome survival and promote tumorigenesis through an increased mutation rate. We showed that oncogenic mutations of Ras, Notch, or Yki enhance the tumorigenic growth potential of *dNKAP*-depleted tissues, resulting in massive tissue overgrowth. Thus, these findings highlight the importance of oncogenic cooperation in tumor transformation and suggest that dNKAP limits the oncogenic potential of active Ras, Notch, or Yki during tumorigenic growth in *Drosophila* wing epithelia. Together, these findings will promote our understanding of how mutations of NKAP family genes cause human cancers and other diseases.

## Materials and methods

### Drosophila stocks

The fly strains used were as follows: *w^1118^, UAS-dNKAP^RNAi^* (Vienna Drosophila RNAi Center, v35065), *UAS-Notch^RNAi^* (THU1994), *UAS-Stat92E^RNAi^* (Bloomington Drosophila Stock Center, BL33637), *UAS-ImpL3^RNAi^*(v31192), *UAS-Dilp8^RNAi^* (BL80436), *UAS-dNKAP, UAS-NICD* (BL52008), *UAS-Ras^V12^, UAS-yki.S168A.GFP.HA* (BL28816), *UAS-Bsk^DN^, UAS-p35, vg(BE)-lacZ, 10×STAT-GFP, puc-lacZ, Su(H)-lacZ, upd-lacZ, ms1096-Gal4;UAS-mCD8-GFP, en-Gal4 UAS-GFP/Cyo, ptc-Gal4 UAS-GFP/Cyo, hh-Gal4 UAS-GFP/TM6B, ap-Gal4 UAS-GFP/Cyo, dNKAP^1^, FRT82B, FRT82B dNKAP^1^/TM6B*, *hsFLP;FRT82B ubi-GFP/TM2*, and *hsFLP;FRT82B/FRT82B Minute ubi-GFP.*

### Immunostaining and EdU labeling

Immunostaining and EdU labeling of wing imaginal discs or salivary glands were performed as described previously. For immunostaining, third-instar larval wing imaginal discs or salivary glands were dissected and fixed for 20 min in phosphate-buffered saline (PBS) (10 mM NaH_2_PO4/Na_2_HPO4, 175 mM NaCl, pH 7.4) with 4% formaldehyde. Fixed samples were washed three times with 0.1% Triton X-100 in PBS (PBT) and blocked in PBT with 3% bovine serum albumin for 1 h at room temperature before incubation with primary antibodies overnight at 4°C. After three washes, samples were then incubated with secondary antibodies and rhodamine-conjugated phalloidin when needed for 2 h. Samples were then incubated with DAPI solution for 20 min. After three washes, samples were mounted in Vectorshield. The primary antibodies used were chicken anti-GFP (1:2000; Abcam, ab13970), mouse anti-β-galactosidase (1:2000; Abcam), mouse anti-armadillo (1:100; DSHB, N2 7A1), mouse anti-MMP1 (1:50; DSHB, 3A6B4), rat anti-E-cadherin (1:100; DSHB, DCAD2-S), mouse anti-NICD (1:100; DSHB, C17.9C6), rabbit anti-cleaved caspase 3 (1:100; Cell Signaling Technology), rabbit anti-aPKC (1:1000, Santa Cruz, sc216), mouse anti-Dlg (1:100, DSHB, 4F3), mouse S9.6 (1:1000, Kerafast, ENH001), mouse anti-γH2Av (1:500; DSHB, Unc93), rabbit anti-γH2Av (1:1000; Rockland, 600-401-914), rabbit anti-Mre11 (1:1000, a gift from Xiaolin Bi), and guinea pig anti-Nbs (1:200, a gift from Yikang Rong). The secondary antibodies used were Alexa Fluor 488-, 555-, or 633-conjugated (anti-rabbit, anti-mouse, or anti-chicken) from Molecular Probes (1:250 or 1:500). Nuclei were stained with DAPI (1 μg/ml, Sigma). For EdU labeling, third-instar larval wing imaginal discs were dissected in Schneider's *Drosophila* (SD) medium and incubated for 30 min in SD medium with 5 μM EdU. Samples were then fixed for 20 min in PBS with 4% formaldehyde. Detection was performed using a Click-iT EdU Alexa Fluor 555 Imaging kit according to the manufacturer's protocol (C10338, Life Technologies). For the TUNEL assay, wing imaginal discs were dissected and fixed as described above. After being permeabilized, the discs were processed using a TUNEL kit according to the manufacturer's protocol. Images were taken on an Olympus FV1000 confocal microscope and processed using Adobe Photoshop.

### Quantification and statistical analysis

For wing imaginal disc size measurement, the size of GFP-positive area in wing imaginal discs was quantified in ImageJ. A color threshold was first set to comprise the entire GFP area in the discs, and the size of GFP-positive area was then determined using the ‘Analyze particles’ option. A similar approach was used to quantify the EdU signal in a defined region. Statistical analysis was performed by unpaired *t*-test with GraphPad Prism 5.

For larval wing disc staining experiments, we did not distinguish biological or technical replicates (e.g. two wing discs per larva). For the control and RNAi experiments, we always used the same setting, including laser intensity and exposure time, when images were captured. All images were processed using ImageJ and Adobe Photoshop. Sample sizes are described in figure legends. For quantifications of the signal intensity in several experiments, data were from the measurements based on three different regions with the same size for each disc as indicated in figure legends.

## Supplementary Material

mjad078_Supplemental_Files

## Data availability

The datasets generated during the current study are available in the Gene Expression Omnibus (GEO) repository at https://www.ncbi.nlm.nih.gov/geo/query/acc.cgi?acc=GSE134531.
