# Supplementary material for: Impaired dNKAP function drives genome instability and tumorigenic growth in Drosophila epithelia
Source: J Mol Cell Biol. 2023 Dec 6;15(12):mjad078. doi: 10.1093/jmcb/mjad078 (PMC11070879; doi:10.1093/jmcb/mjad078)
Supplement: mjad078_Supplemental_Files [file mjad078_supplemental_files.zip › JMCB-2023-0053-R1 Supplementary data.pdf]

Supplementary Materials and Methods

Generation of UAS-dNKAP transgenic flies

To generate the *UAS-dNKAP* transgene, a full length cDNA of *dNKAP* was amplified with the primers, and cloned into the *pUAST-attB* vector. The primers used were as follows: 5'-GCGGCCGCATGCGTTCACGCAGCAGGAGTC-3' and 5'-TCTAGATTACTTCTTGTCCTTGGCCTGC-3'. The obtained *pUAST-attB-dNKAP* construct was transformed into embryos carrying *M{vas-int.Dm}ZH-2A; P{CaryP}attP40* through site specific integration.

Clonal analysis

Mitotic clones in wing imaginal discs were induced using the FLP/FRT system. For generation of dNKAP mutant clones, embryos were collected within 2 hr after egg laying (AEL). Hatched larvae were heat shocked at 37 °C for 1 hr at 34-36 hr AEL or at 70-72 hr AEL. Wing imaginal discs were dissected and fixed at 122-124 hr AEL. For generation of dNKAP and minute mutant clones, embryos were collected within 12 hr AEL and hatched larvae were heat shocked for 1 hr at 48-60 hr AEL. Dissection were done at wondering larval stage (around 120 hr).

Measurement of the developmental timing of pupariation

For measurement of the developmental timing of pupariation, embryos were collected within 6 hr AEL. 24 hr later, 30 L1 hatched larvae were collected and reared per tube. The number of pupae at a given time was scored every 8 hours. Data are from three independent experiments.

Mutant generation

The $dNKAP^1$ mutant allele was obtained through ends-out homologous recombination using the pW25-RMCE vector as described. Homologous regions flanking the 5' and 3' parts of dNKAP (CG6066) were amplified and cloned into the pW25-RMCE vector. Primer pairs used are as the following:

Upstream: 5'-ACAGGGTAATGTACCGCGCTGTTGCTGGAACTTGGAGCAC-3' and 5'-

GTACCGCATGCGCGGCCGTGGAAAATACAAGTTTTTAAC-3'

Downstream: 5'-CCTGCAGGATCCTAAAGGATATTGTCTTC-3' and 5'-CCTGCAGGATTCGCGAATATTAAAACAATATTG-3'

Ends-out homologous recombination results in the replacement of the entire dNKAP coding sequence with the mini-white marker gene. Homologous recombination was confirmed by genomic PCR using the following primer pairs:
Upstream: 5'-GCATTCTTCTCGTTCATCAGG-3' and 5'-AGTGAGAGAGCAATAGTACAGAGAGG-3'
Downstream: 5'-GCAAACACAATCACACAAATGTGC-3' and 5'-GTCTCGTGAAGTTCTCCACG-3'

The presence or absence of dNKAP transcripts was examined by RT-PCR using the following primers that would amplify the dNKAP coding region: 5'-CTAACATCCGATGAAATCGC-3' and 5'-CTTCTTGTCCTTGGCCTG-3'.

RNA in situ hybridization

Digoxigenin-labeled RNA probes were synthesized from the PCR products using RNA polymerase T7 and primers with T7 sequence in their ends. PCR primers used were as follows: GCTGGTCATCGGAGTCTGTTGCCT and TGTTGTAGGACCTGCTCGAGTGAT for Dilp8, and GGTGTGCCTCATCGATGTCT and CACTTCTCTGGATCCTCGCC for ImpL3. Third instar larval wing imaginal discs were dissected in cold PBS and fixed in PBT (1x PBS with 0.1%(vol/vol) Tween-20) with 4% paraformaldehyde for 20 min at room temperature. After several washes, discs were permeabilized with 1% Triton X-100 in PBT for 1 hour followed by three washes and then quenched with 1% H_2O_2 in PBT for 10 min. Discs were dehydrated through a serious of ethanol solutions diluted in PBT and then stored at -20 °C. Subsequently, the samples were rehydrated and then re-fixed for 30 min in PBT with 4% paraformaldehyde. After three washes, discs were pre-hybridized for 1 hour at 60 °C in HYB solution, and hybridized with the RNA probes overnight at 60 °C. The RNA probes were diluted at a dilution of 1/500 and denatured for 10 min at 68 °C. After hybridization, samples were washed in HYB and PBT and then blocked for 1 h in WBR/PBT buffer. Samples were then incubated for 2 h at room temperature in a 1:2000 dilution of anti-DIG

antibody in WBR/PBT buffer. After incubation, the probes were detected using NBT/BCIP solution (Roche, cat. no. 11681451001). Discs were mounted in 80% glycerol. Images were acquired on a Nikon Eclipse 80 microscope and processed using Adobe Photoshop.

RNA fluorescence in situ hybridization (FISH) and antibody staining

Alexa Fluor® 594 labeled RNA probes were synthesized by using FISH Tag™ RNA Multicolor Kit (Invitrogen, F32956) according to manufacturer's instructions. The same primers for RNA in situ hybridization were used for Dilp8. Third instar larval wing imaginal discs were dissected, fixed and permeabilized as the same as described for RNA in situ hybridization. Samples were then blocked with blocking buffer (1x PBT with 5% BSA) for 1h followed by incubation with primary antibodies (chicken anti-GFP, 1:1000, Invitrogen) overnight at 4 °C. After three times of PBT washes, samples were incubated with secondary antibodies (anti-chicken 488, 1:500, Jackson) for 2 h. Samples were washed again three times and then fixed with 4% paraformaldehyde for 30 min. Subsequently, samples were treated with Proteinase K (1:5000 in PBS) and washed three times. After that, samples were pre-hybridized, hybridized with the RNA probes and washed as the same as described for RNA in situ hybridization. Samples were then mounted in Vectorshield. Images were obtained using an Olympus FV1000 confocal microscope.

RNA-Sequencing and splicing analysis

RNA extractions was performed using Trizol from 70 wandering third instar larval wing imaginal discs. 1 ug total RNA was sent to Personalbio (Personalbio, Shanghai, China) for sample preparation and sequencing. Three pools of imaginal discs were made from both the control (*MS1096-Gal4*) and dNKAP knockdown (*MS1096-Gal4>dNKAP*^{RNAi}) larvae. Briefly, total RNA libraries were prepared according to the Illumina protocol and sequenced on an Illumina NextSeq 500 platform, using paired-end chemistry with 2 × 150 bp read lengths (Illumina).

More than 40 million reads were generated for each sample. Basic reads quality check was performed with FastQC. Reads were mapped to the Drosophila melanogaster reference genome assembly (version dm6.09, 2016) using Tophat2.0 and gene quantification was carried out using HTseq. Differential expression analysis was performed using DEseq. Genes with a p-value<0.05 (q-value<0.05) and fold change>2 in DEseq analysis were defined as differentially expressed genes. Gene ontology (GO) functional classification of differentially expressed was performed using the map2slim program. The p value for GO enrichment analysis was calculated using the hypergeometric test. Differential inclusion rates across 10400 splicing events between *dNKAP* knockdown and control discs were calculated using rMATS (v4.0.1). Splicing events with inclusion differences ≥ 10% and FDR ≤ 0.01 were considered significantly altered. Pathway analysis for alternatively spliced genes was performed using clusterProfiler (v4.6.0).

RT-qPCR and RT-PCR

For each biological replicate, total RNA was isolated from about fifty third instar larval wing imaginal discs with TRIzol (Invitrogene) reagent. cDNA was synthesized from 1 ug of RNA using oligo-dT primers and SuperScript III (Life technologies). Quantitative PCR was performed in triplicate with the Power SYBR® Green PCR Master Mix (Applied Biosystems) and the ABI 7900HT Fast Real-Time PCR System. Primers used were listed in Table S4. Data were normalized to rp49 transcript levels. RT-PCR was carried out according to the standard protocols from Ambion.

Adult wing analysis

Adult female wings were mounted in 80% glycerol. Images were taken on a Nikon Eclipse 80 microscope and processed using Adobe Photoshop.

Supplementary Figures and Tables

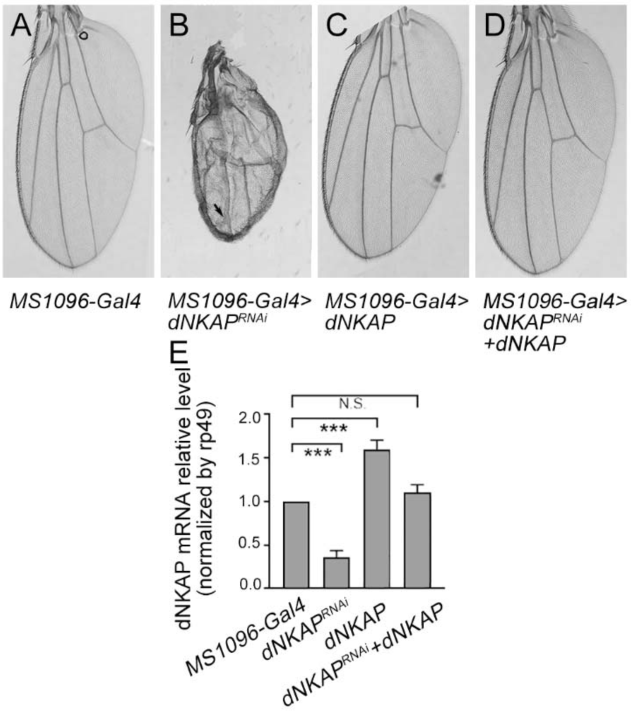

Figure S1 *dNKAP* knockdown causes abnormal wing morphology and growth defects. (A-D) Wings from *MS1096-Gal4* (A), *MS1096-Gal4* driving *dNKAP^{RNAi}*(B), *MS1096-Gal4* driving *UAS-dNKAP* (C) or *MS1096-Gal4* driving *dNKAP^{RNAi}* and *UAS-dNKAP* (D) adult flies. Arrow indicates extra vein tissues. (E) RNAi of *dNKAP* by *MS1096-Gal4* reduces dNKAP transcript levels. *** $p<0.001$.

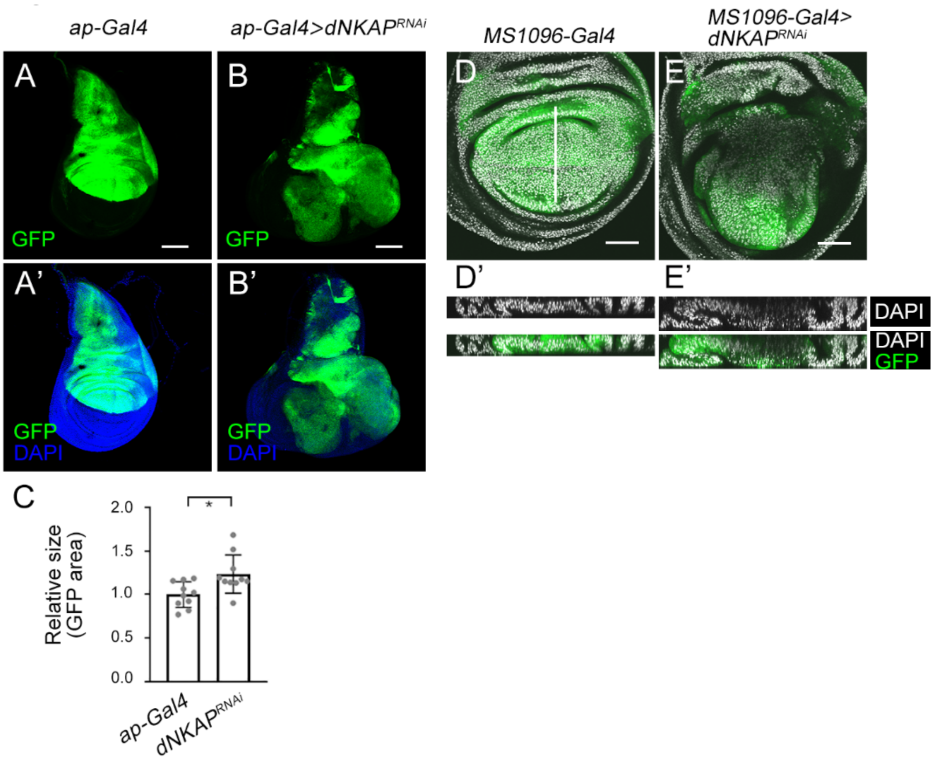

Figure S2 Tissue overgrowth and disorganization of wing discs upon dNKAP knockdown. (A-B') Knockdown of *dNKAP* causes tissue overgrowth. Wing imaginal discs from *ap-Gal4* driving *UAS-GFP* control (A-A') or *ap-Gal4* driving *UAS-GFP* and *UAS-dNKAP*^{RNAi} (B-B') larvae were stained with DAPI and anti-GFP. (C) Quantification of GFP positive area in wing discs with indicated genotypes. n=10. (D-E') Knockdown of dNKAP causes disorganization of wing discs. Wing imaginal discs from *MS1096-Gal4* driving *UAS-mCD8-GFP* control (D-D') or *MS1096-Gal4* driving *UAS-mCD8-GFP* and *UAS-dNKAP*^{RNAi} (E-E') larvae were stained with DAPI and anti-GFP. Panels A-B' and D-E show single confocal sections of third instar wing imaginal discs with dorsal side up. Shown in panels D' and E' are the optical cross-sections of the wing imaginal disc with apical side up. Scale Bars: 100 μm in A and B; 50 μm in D and E.

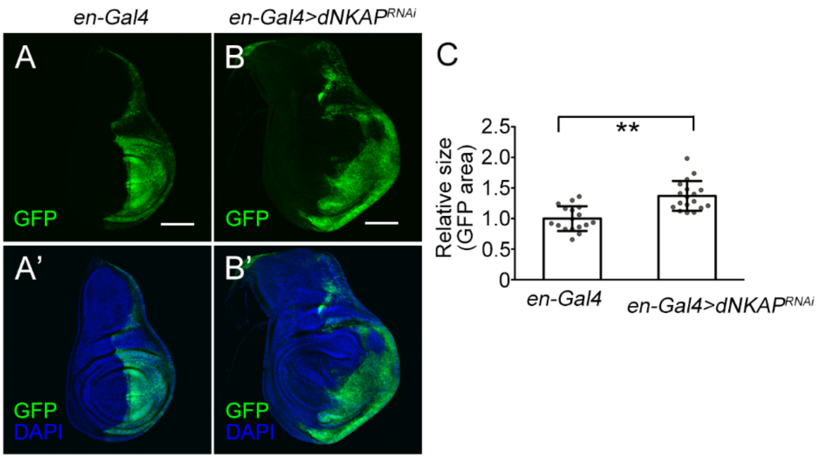

Figure S3 Increased tissue size in *dNKAP* knockdown wing discs. Wing imaginal discs from *en-Gal4* driving *UAS-GFP* control (A-A') or *en-Gal4* driving *UAS-GFP* and *UAS-dNKAP*^{RNAi} (B-B') larvae were stained with DAPI and anti-GFP. (C) Quantification of GFP positive area in both the control and *dNKAP* knockdown wing discs. n=17 and 18, respectively. The value was calculated by normalizing to the mean area of the control disc. Panels A-B' show single confocal sections of third instar wing imaginal discs with posterior side to the right and dorsal side up. Scale Bars: 100 μm. ** $p<0.01$.

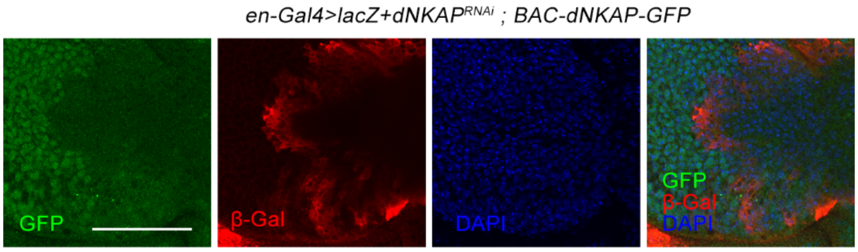

Figure S4 Reduced BAC-dNKAP-GFP levels upon *dNKAP* knockdown. Wing imaginal discs from *en-Gal4* driving *UAS-lacZ* and *UAS-dNKAP*^{RNAi} larvae carrying a *BAC-dNKAP-GFP* transgene were stained with DAPI, anti-β-galactosidase and anti-GFP. All panels show single confocal sections of third instar wing imaginal discs with posterior side to the right and dorsal side up. Scale Bars: 100 μm.

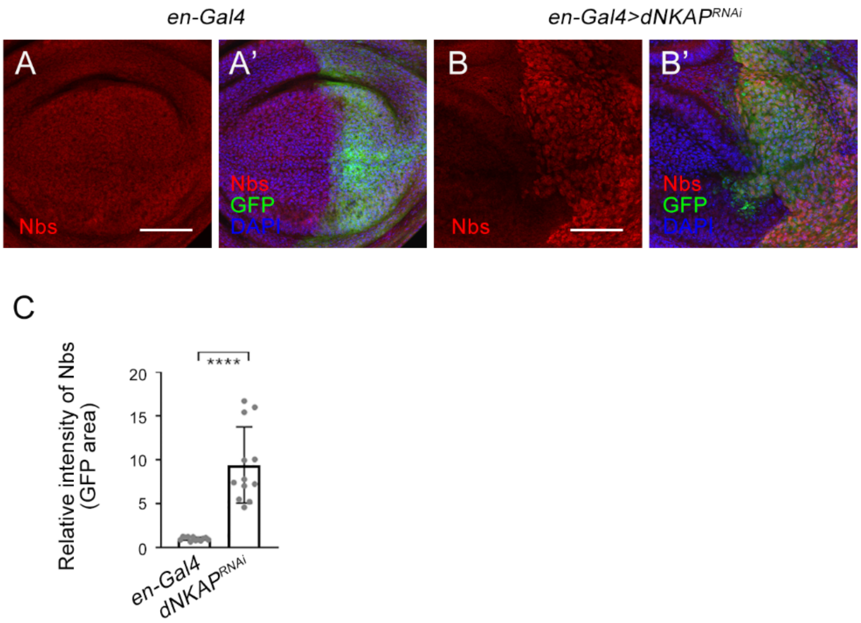

Figure S5 Increased Nbs protein levels in *dNKAP* deficient wing disc cells. (A-B') Wing imaginal discs from *en-Gal4* driving *UAS-GFP* control (A and A') or *en-Gal4* driving *UAS-GFP* and *UAS-dNKAP*^{RNAi} (B and B') larvae were stained with DAPI, anti-Nbs and anti-GFP. All panels show single confocal sections of third instar wing imaginal discs with posterior side to the right and dorsal side up. (C) Quantification of Nbs signals from A and B. n=12, Note that three different regions with the same size were calculated per disc. Scale Bars: 50 μm.

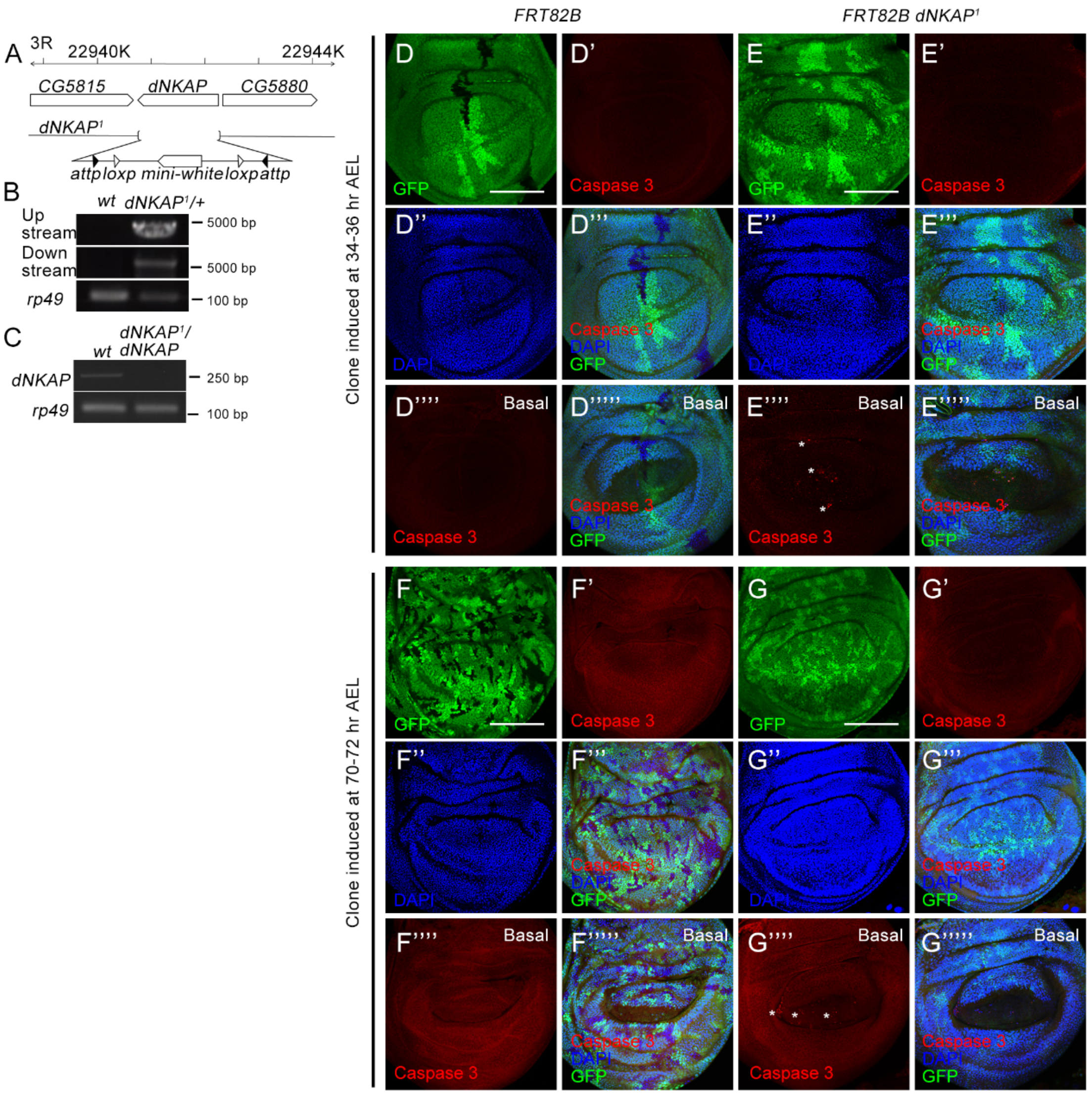

Figure S6 Generation and characterization of *dNKAP* loss-of-function mutants. (A) Diagrams of the dNKAP locus and *dNKAP¹* mutation. The dNKAP coding region was replaced with the mini-white gene by ends-out homologous recombination. (B) PCR examinations of genomic DNAs of *dNKAP¹* homozygous mutants and controls. Primers for checking the left arm and the right arm were listed in the materials and methods section. (C) RT-PCR showing no detectable dNKAP transcript in *dNKAP¹* homozygous mutants. (D-G’’’’’) *dNKAP¹* mutant clones exhibit a growth defect in wing discs. Clones (marked by absence of GFP) of wild type or *dNKAP¹* mutant cells are shown in wing discs from third instar larvae. Clones were induced at 34-36 hr AEL (D-E’’’’’) or 70-72 hr AEL (F-G’’’’’) as indicated. There are no *dNKAP¹* homozygous mutant clones (E and G), whereas clones of a wild type chromosome and their corresponding twin-spots are comparable (D and F). Ectopic caspase 3 signals were observed on the basal surface of wing discs when *dNKAP¹* mutant clones were induced. Wing imaginal discs were stained with anti-GFP, anti-caspase 3 and DAPI. Panels D-G’’’’’ show single confocal sections of third instar wing imaginal discs with dorsal side up. Genotypes: (D-D’’’’’ and F-F’’’’’), *hs*FLP; FRT82B/FRT82B *ub*-GFP, (E-E’’’’’ and G-

G’’’’’), *hs*FLP; FRT82B *dNKAP*[1]/FRT82B *ub-GFP*. Scale bars: 100 μm.

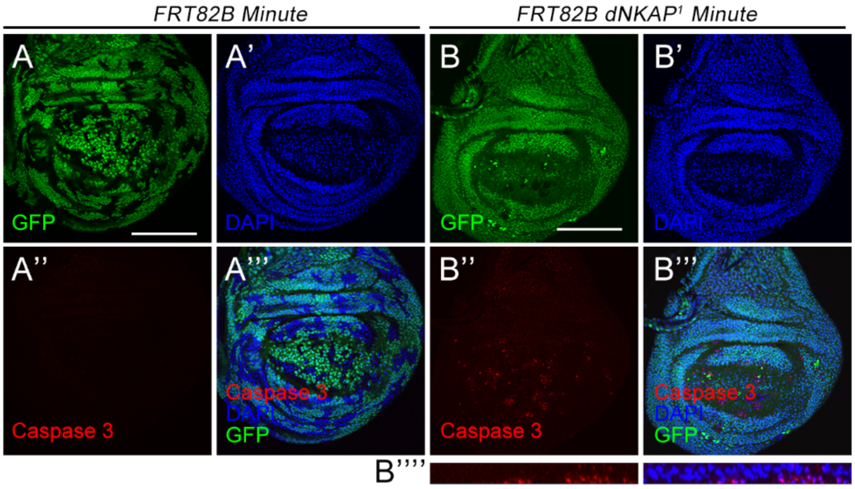

Figure S7 High cell death levels in small *dNKAP*[1] homozygous mutant clones in a minute background. (A-B’’’’) Clones (marked by absence of GFP) of wild type or *dNKAP*[1] mutant cells in a *Minute (M)* mutant background are shown in wing discs from third instar larvae. Wing imaginal discs were stained with anti-caspase 3, anti-GFP and DAPI. Panels A-B’’’ show single confocal sections of third instar wing imaginal discs with dorsal side up. Shown in B’’’’ is the optical cross-section of the wing imaginal disc with apical side up. Genotypes: (A-A’’’), *hs*FLP; FRT82B/FRT82B Minute ubi-GFP, (B-B’’’’), *hs*FLP; FRT82B *dNKAP*[1]/FRT82B Minute ubi-GFP. Scale bars: 100 μm.

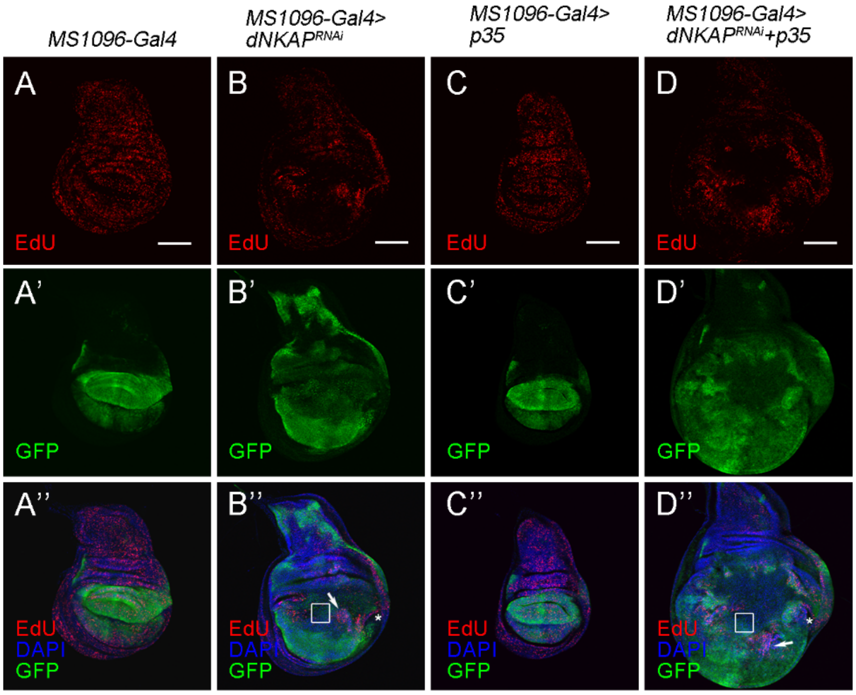

Figure S8 Aberrant cell proliferation in *dNKAP* knockdown wing discs. (A-D’)

Decreased EdU signal in a population of *dNKAP* depletion cells within the wing pouch region. Wing imaginal discs from *MS1096-Gal4* driving *UAS-GFP* control (A-A''), *MS1096-Gal4* driving *UAS-GFP* and *UAS-dNKAP^{RNAi}* (B-B''), *MS1096-Gal4* driving *UAS-GFP* and *UAS-p35* (C and C'') or *MS1096-Gal4* driving *UAS-GFP*, *UAS-dNKAP^{RNAi}* and *UAS-p35* (D and D'') larvae were labeled with EdU and stained with DAPI and anti-GFP. Boxes in B'' and D'' indicate the pouch region. Note EdU signals are increased in a patch of *dNKAP* depletion cells (Indicated by the arrows in B'' and D'') and wild type cells (indicated by the stars in B and D) adjacent to *dNKAP* depletion cells. Scale bars: 100 μm.

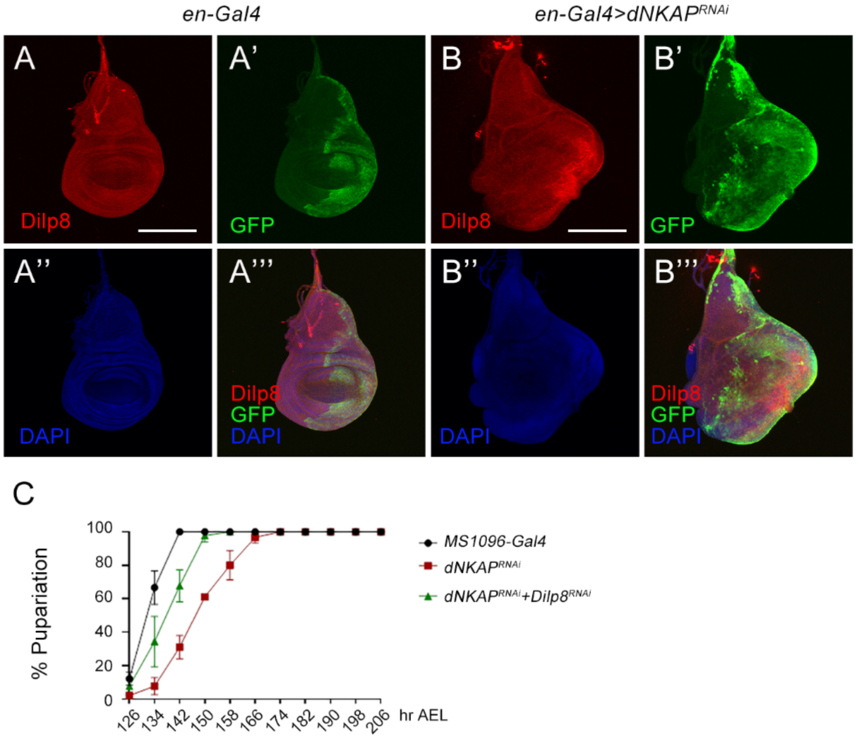

Figure S9 Upregulation of Dilp8 and its effect on developmental delay in *dNKAP* depletion wing discs. (A-B''') Increased *Dilp8* mRNA level in *dNKAP* knockdown wing discs. Wing imaginal discs from *en-Gal4* driving *UAS-GFP* control (A-A''') or *en-Gal4* driving *UAS-GFP* and *UAS-dNKAP^{RNAi}* (B-B''') larvae were labeled with a fluorescein-labeled *Dilp8* RNA probe and stained with anti-GFP and DAPI. (C) Partial rescue of the developmental delay upon Dilp8 depletion in dNKAP knockdown larvae. Data showing the percentage of larvae that have pupariated at the indicated time (AEL).

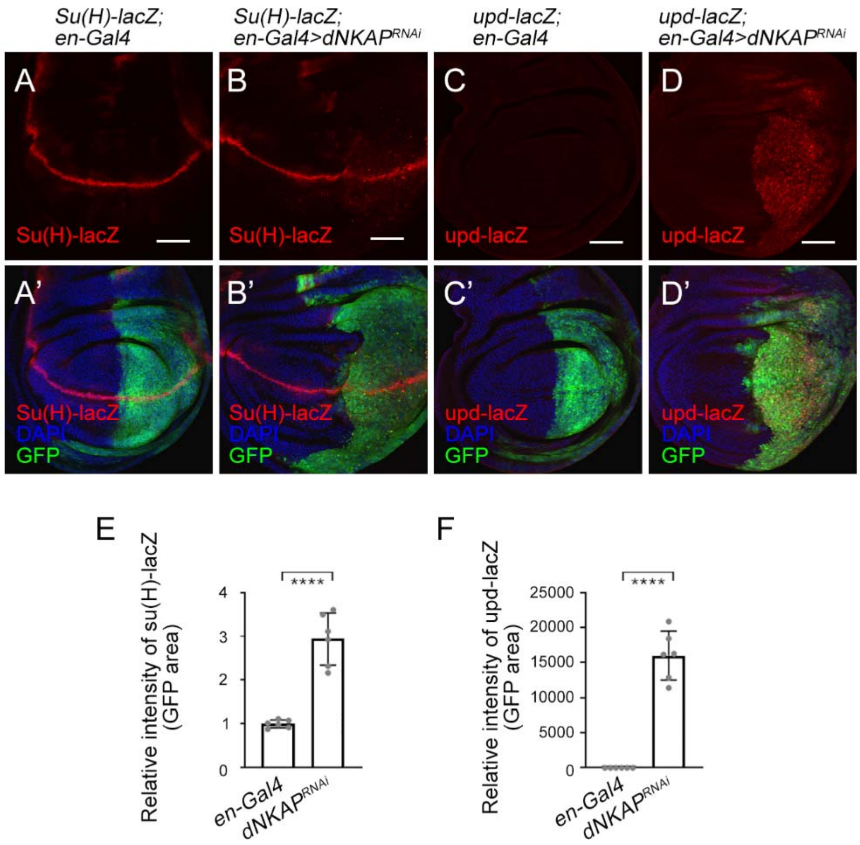

Figure S10 Activation of Notch and JAK/STAT signaling in *dNKAP* depletion wing discs. (A-B') Upregulation of *Su(H)-lacZ* in *dNKAP* knockdown wing discs. Wing imaginal discs from *en-Gal4* driving *UAS-GFP* control (A and A') or *en-Gal4* driving *UAS-GFP* and *UAS-dNKAP*^{RNAi} (B and B') larvae carrying a *Su(H)-lacZ* transgene were stained with DAPI, anti-β-galactosidase and anti-GFP. (C-D') Upregulation of *upd-lacZ* in *dNKAP* knockdown wing discs. Wing imaginal discs from *en-Gal4* driving *UAS-GFP* control (C and C') or *en-Gal4* driving *UAS-GFP* and *UAS-dNKAP*^{RNAi} (D and D') larvae carrying an *upd-lacZ* transgene were stained with DAPI, anti-β-galactosidase and anti-GFP. GFP was used to mark the knockdown domain. All panels show single confocal sections of third instar wing imaginal discs with posterior side to the right and dorsal side up. (E) Quantification of Su(H)-lacZ signals from A and B. n=6, Note that three different regions with the same size were calculated per disc. (F) Quantification of upd-lacZ signals from C and D. n=6, Note that three different regions with the same size were calculated per disc. Scale Bars: 50 μm.

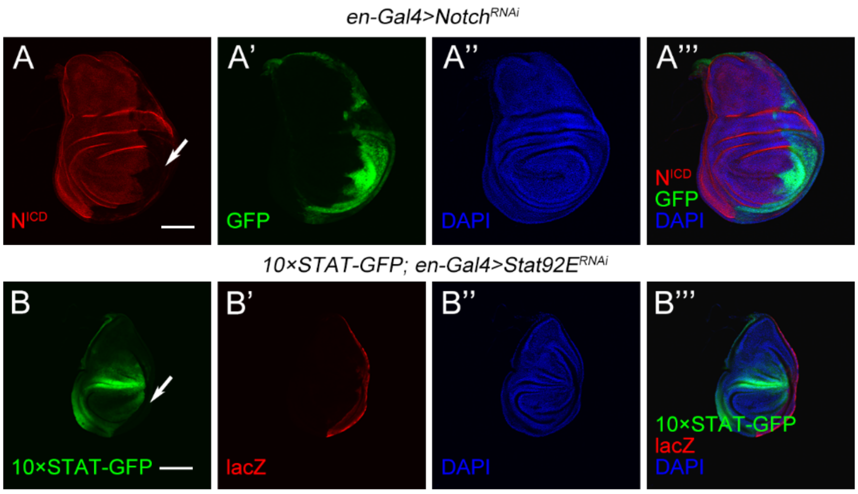

Figure S11 Specificity of both *Notch* and *Stat92E* RNAi lines. (A-A''') Reduction of Notch protein level upon Notch depletion. Wing imaginal discs from *en-Gal4* driving *UAS-GFP* and *UAS-Notch*^{RNAi} larvae were stained with DAPI, anti-N^{ICD} and anti-GFP. (B-B''') Downregulation of 10XSTAT-GFP upon *Stat92E* depletion. Wing imaginal discs from *en-Gal4* driving *UAS-GFP* and *UAS-Stat92E*^{RNAi} larvae were stained with DAPI, anti-GFP and anti-β-galactosidase and. GFP or lacZ was used to mark the knockdown domain. All panels show single confocal sections of third instar wing imaginal discs with posterior side to the right and dorsal side up. Scale Bars: 100 μm.

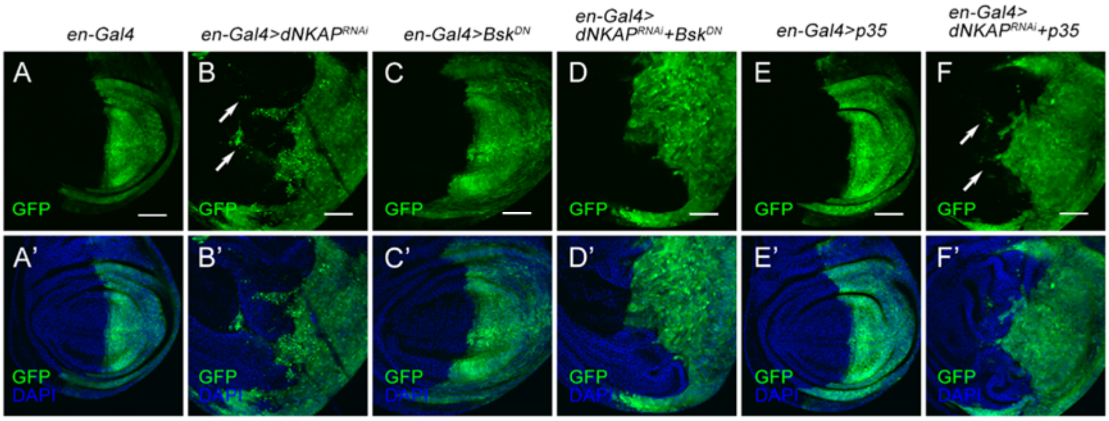

Figure S12 JNK signaling is required for cell invasiveness in *dNKAP* knockdown tissues. (A-D') Inhibition of JNK signaling suppresses cell invasive behavior in *dNKAP* knockdown wing discs. Wing imaginal discs from *en-Gal4* driving *UAS-GFP* control (A and A'), *en-Gal4* driving *UAS-GFP* and *UAS-dNKAP*^{RNAi} (B and B'), *en-Gal4* driving *UAS-GFP* and *UAS-Bsk*^{DN} (C and C') or *en-Gal4* driving *UAS-GFP, UAS-Bsk*^{DN} and *UAS-dNKAP*^{RNAi} (D and D') larvae were stained with DAPI and anti-GFP. (E-F') Inhibition of apoptosis does not suppress cell invasive behavior *dNKAP* knockdown wing discs. Wing imaginal discs from *en-Gal4* driving *UAS-GFP* and *UAS-p35* (E and E') or *en-Gal4* driving *UAS-GFP, UAS-p35* and *UAS-dNKAP*^{RNAi} (F and F') larvae were stained with DAPI and anti-GFP. GFP positive cells migrates

toward the anterior compartment (Indicated by the arrows). GFP was used to mark the knockdown domain. All panels show single confocal sections of third instar wing imaginal discs with posterior side to the right and dorsal side up. Scale Bars: 50 μm.

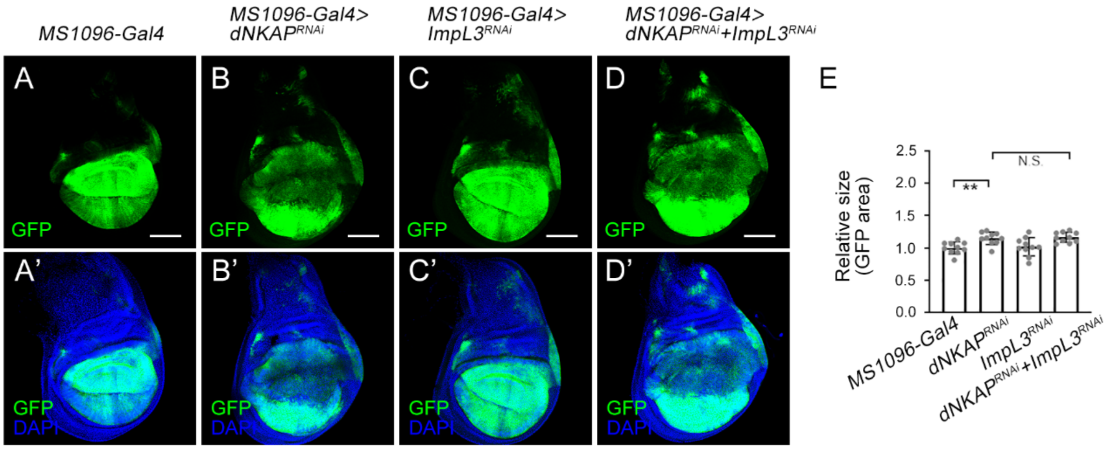

Figure S13 Knockdown of ImpL3 does not rescue the tumorigenic growth in dNKAP depletion wing discs. (A-D') Wing imaginal discs from *MS1096-Gal4* driving *UAS-mCD8-GFP* control (A and A'), *MS1096-Gal4* driving *UAS-mCD8-GFP* and *UAS-dNKAP*^{RNAi} (B and B'), *MS1096-Gal4* driving *UAS-mCD8-GFP* and *UAS-ImpL3*^{RNAi}(C and C') and *MS1096-Gal4* driving *UAS-mCD8-GFP, UAS-ImpL3*^{RNAi} and *UAS-dNKAP*^{RNAi} (D and D') larvae were stained with DAPI and anti-GFP. (E) Quantification of GFP positive area in wing discs with indicated genotypes. n=10 for each genotype. Scale bars: 100 μm.

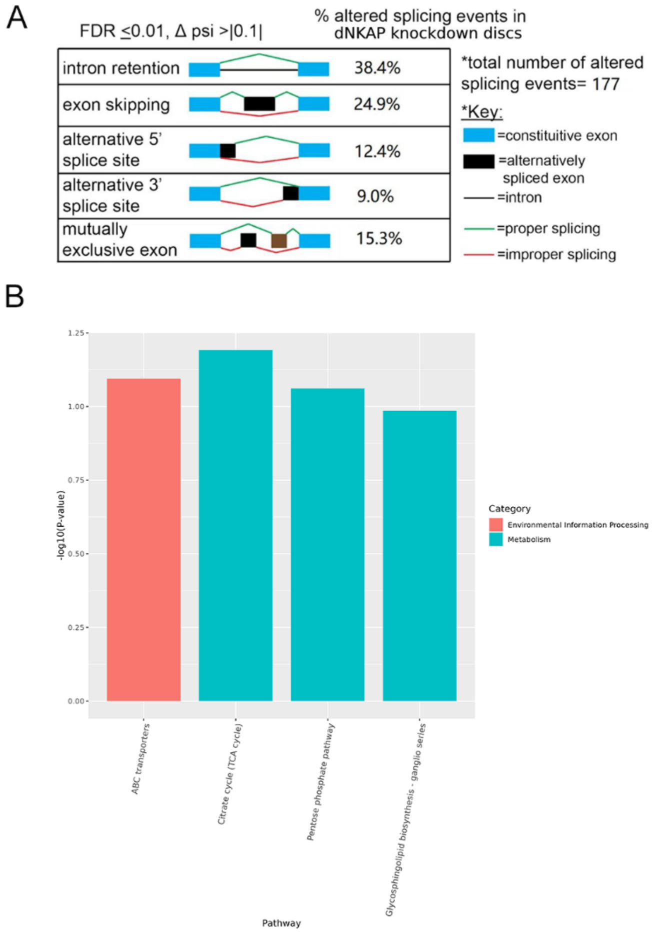

Figure S14 dNKAP knockdown results in mRNA splicing defects. (A) Schematic summary of different splicing events analyzed and quantification of different splicing events altered in *dNKAP* knockdown wing discs. (B) Pathways enriched in alternatively spliced genes in dNKAP knockdown wing discs compared to controls.

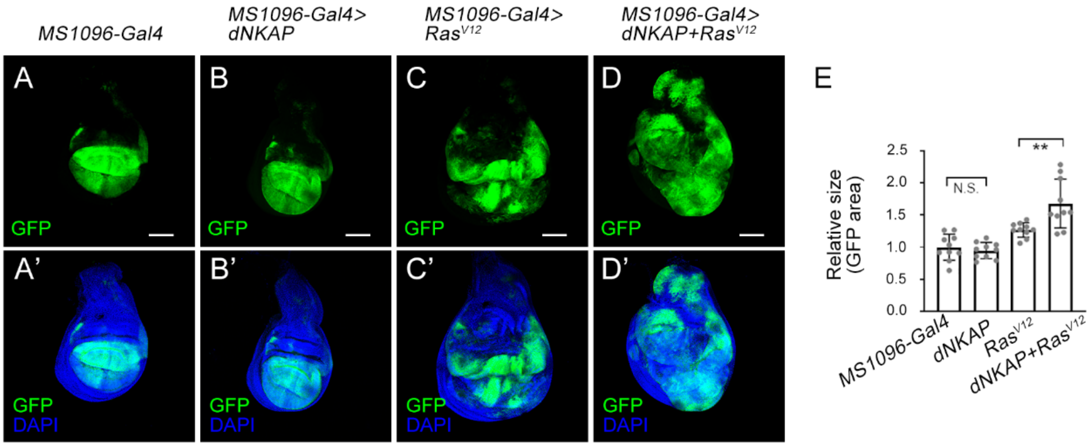

Figure S15 Overexpression of dNKAP promotes tumorigenic growth in cooperation with oncogenic Ras^{V12}. (A-D') Wing imaginal discs from *MS1096-*

Gal4 driving *UAS-mCD8-GFP* control (A and A'), *MS1096-Gal4* driving *UAS-mCD8-GFP* and *UAS-dNKAP* (B and B'), *MS1096-Gal4* driving *UAS-mCD8-GFP* and *UAS-Ras^{v12}*(C and C') and *MS1096-Gal4* driving *UAS-mCD8-GFP, UAS-dNKAP* and *UAS-Ras^{v12}* (D and D') larvae were stained with DAPI and anti-GFP. (E) Quantification of GFP positive area in wing discs with indicated genotypes. n=10 for each genotype. Scale bars: 100 μm.

Table S1 Overexpression of *dNKAP* rescues the lethality phenotype of in *dNKAP¹* mutants

Cross: UAS-dNKAP/Cyo;dNKAP¹/TM2 x Tub-GAL4/Cyo;dNKAP¹/TM6B	
Genotypes of progeny	Number
Tub-GAL4/UAS-dNKAP; dNKAP¹/dNKAP¹	5
Tub-GAL4/UAS-dNKAP; dNKAP¹/TM2	9
Tub-GAL4/UAS-dNKAP; dNKAP¹/TM6B	5
Tub-GAL4/UAS-dNKAP; TM2/TM6B	4
Tub-GAL4 (OR UAS-dNKAP)/Cyo; dNKAP¹/dNKAP¹	0
Tub-GAL4 (OR UAS-dNKAP)/Cyo; dNKAP¹/TM2	27
Tub-GAL4 (OR UAS-dNKAP)/Cyo; dNKAP¹/TM6B	12
Tub-GAL4 (OR UAS-dNKAP)/Cyo; TM2/TM6B	12

Table S2 Upregulated and downregulated gene lists in dNKAP knockdown tissues as compared to the control

A.upreulgated gene list

Gene Symbol	id	baseMean	baseMean Control	baseMean dNKAP RNAi	foldChange
CG14332	FBgn0038509	280.4880864	0.251158313	560.7250145	2232.556067
CG42834	FBgn0262023	151.6067236	0.585198807	302.6282483	517.1375007
Ilp8	FBgn0036690	762.2463437	9.201235741	1515.291452	164.6834723
CG15431	FBgn0031602	343.8346966	5.448352738	682.2210405	125.2160191
Eglp2	FBgn0034883	10.55677991	0.251158313	20.8624015	83.06474624
CG4269	FBgn0034741	10.583544	0.334040494	20.8330475	62.36683238
CG17237	FBgn0031410	10.50255329	0.352351376	20.65275521	58.61408977
Mal-A6	FBgn0050360	14.05838049	0.502316625	27.61444436	54.97417958
CG5955	FBgn0036997	92.04978973	3.49904242	180.600537	51.61427481
CG42319	FBgn0259219	711.5029083	29.38532033	1393.620496	47.42573776
CG14695	FBgn0037850	250.0319366	10.35332247	489.7105507	47.29984524
Arc1	FBgn0033926	8375.726552	349.4721409	16401.98096	46.93358652
CG10799	FBgn0033821	5.71406629	0.251158313	11.17697427	44.50170951
CG6870	FBgn0032652	29.90532126	1.338673745	58.47196878	43.67902861
CG4271	FBgn0031409	5.244433994	0.251158313	10.23770968	40.76197825
RabX2	FBgn0030200	51.27545126	2.579803119	99.97109941	38.75144528
Ada1-1	FBgn0051865	42.47570331	2.20914086	82.74226576	37.45449973
CG14528	FBgn0039611	11.39474566	0.668080988	22.12141033	33.11186925
IM33	FBgn0031561	9.952501372	0.585198807	19.31980394	33.01408635
CG32625	FBgn0052625	35.86185292	2.144569561	69.57913627	32.4443364
AOX1	FBgn0267408	972.0486539	58.29228388	1885.805024	32.35085158
CG8620	FBgn0040837	169.8668494	10.55822037	329.1754785	31.17717447
Cyp6a20	FBgn0033980	10.60836598	0.668080988	20.54865097	30.75772448
CG10827	FBgn0038845	5.283544164	0.352351376	10.21473695	28.99020023
fd19B	FBgn0031086	4.945657691	0.352351376	9.538964006	27.07230522
Npc2h	FBgn0039801	4.679747295	0.334040494	9.025454095	27.01904187
CG2064	FBgn0033205	3088.252585	227.5267852	5948.978384	26.14627715
slbo	FBgn0005638	33.57214045	2.478610055	64.66567085	26.08948943
Sardh	FBgn0034276	12.25420807	0.919239301	23.58917684	25.66162785
robl22E	FBgn0028570	4.417289564	0.334040494	8.500538635	25.44762921
CG13315	FBgn0040827	16.40393482	1.253279795	31.55458984	25.17760995
GstE6	FBgn0063494	680.8675858	53.64576984	1308.089402	24.3838313
drd	FBgn0260006	409.7431047	34.23598284	785.2502266	22.93640087
CG4306	FBgn0036787	3.922487417	0.334040494	7.510934339	22.48510127
CG33509	FBgn0053509	10.62656944	0.919239301	20.33389958	22.12035491
CG42587	FBgn0260955	17.57900924	1.52274899	33.63526948	22.08851866
Odc1	FBgn0013307	7.755769215	0.68639187	14.82514656	21.5986628
CG8745	FBgn0036381	85.63498635	7.727088433	163.5428843	21.16487804
CG16926	FBgn0040732	55.23864086	5.114312244	105.3629695	20.60159108
stops	FBgn0086704	13.60989267	1.271590677	25.94819466	20.40609068
Fbp1	FBgn0000639	9072.932315	855.764501	17290.10013	20.20427362
Gbp3	FBgn0039031	29.25494355	2.858910966	55.65097613	19.46579547
CG15784	FBgn0029766	9282.02362	926.7509276	17637.29631	19.03132308
CG7080	FBgn0038941	23.32186201	2.478610055	44.16511397	17.81850028
Tsp42Ed	FBgn0029507	126.7514688	13.60006965	239.9028679	17.63982641
Sodh-2	FBgn0022359	28.08610981	3.017548445	53.15467117	17.61518403

CG12699	FBgn0046294	14.68036142	1.605631171	27.75509167	17.2860942
Shawl	FBgn0085395	8.51649036	1.002121482	16.03085924	15.99692205
upd3	FBgn0053542	356.9950512	42.92994583	671.0601566	15.6315165
CG2065	FBgn0033204	198.9843522	24.13130691	373.8373974	15.49180071
CG18067	FBgn0034512	26.50457195	3.229573225	49.77957068	15.41366837
Osi15	FBgn0037424	40.8165712	4.975530993	76.6576114	15.40692069
CG33307	FBgn0053307	5.214807734	0.668080988	9.761534479	14.61130409
GstE7	FBgn0063493	46.4084016	5.956829825	86.85997338	14.5815771
CG2909	FBgn0030189	426.7926531	54.8972307	798.6880755	14.54878626
Cyp6d5	FBgn0038194	18.14311439	2.340795227	33.94543356	14.50166728
CG33120	FBgn0053120	4.591871013	0.603509689	8.580232337	14.21722384
CG32440	FBgn0052440	50.97945374	6.746926527	95.21198096	14.11190422
CG3397	FBgn0037975	183.7884911	24.51411959	343.0628626	13.99450065
JhI-26	FBgn0028424	42.64177746	5.744805045	79.53874987	13.84533492
CG13796	FBgn0031939	6.956760779	0.937550183	12.97597137	13.84029528
Arc2	FBgn0033928	544.8150582	73.54583696	1016.084279	13.81566002
ppk13	FBgn0053508	279.0442722	37.8016999	520.2868445	13.76358327
Idgf5	FBgn0064237	9.693535083	1.336161976	18.05090819	13.50952093
DAT	FBgn0034136	26.56177927	3.667318551	49.45623999	13.48566788
CG42370	FBgn0259716	20.37527453	2.843111852	37.9074372	13.3330798
CG8586	FBgn0033320	9.105461347	1.271590677	16.93933202	13.32137166
Swim	FBgn0034709	654.117447	91.4263735	1216.80852	13.30916314
Fbp2	FBgn0000640	568.4831456	80.04278859	1056.923503	13.20448127
Obp99c	FBgn0039682	139.9655046	19.9452937	259.9857154	13.03494044
CG2233	FBgn0029990	256.4962455	36.82353826	476.1689528	12.93110264
Tps1	FBgn0027560	52.42845981	7.742887548	97.11403207	12.54235341
CG3492	FBgn0035007	18.72624922	2.800500129	34.65199832	12.37350356
CG3999	FBgn0037801	22.69909389	3.444109773	41.954078	12.18139977
Cyp28d1	FBgn0031689	21.38892371	3.27834541	39.49950201	12.048609
CG34166	FBgn0085195	492.3141876	75.48263493	909.1457402	12.04443566
Cyt-b5-r	FBgn0000406	13.58442108	2.089636914	25.07920524	12.00170473
upd2	FBgn0030904	254.2598086	40.62074883	467.8988684	11.51871597
bru3	FBgn0264001	49.52494719	7.991534092	91.05836028	11.39435298
CG13607	FBgn0039151	31.37579453	5.114312244	57.63727683	11.26980014
CG32437	FBgn0052437	135.287802	22.10008083	248.4755232	11.24319522
Ir7g	FBgn0029968	4.967702631	0.854668002	9.08073726	10.62487099
CG4725	FBgn0039022	7.725315915	1.338673745	14.11195809	10.54174562
Cyp6d2	FBgn0034756	261.3320691	46.52436186	476.1397763	10.23420327
CG1468	FBgn0030157	5.056030758	0.919239301	9.192822216	10.00046692
CG12116	FBgn0030041	68.43450028	12.60256329	124.2664373	9.860409699
CG3494	FBgn0035008	5.551492573	1.057054129	10.04593102	9.503705383
CG11897	FBgn0039644	1366.34663	260.2275633	2472.465696	9.501167613
psh	FBgn0030926	98.71864803	18.91425626	178.5230398	9.438543994
Prat2	FBgn0041194	56.58751719	10.98461119	102.1904232	9.30305328
alpha-Est7	FBgn0015575	33.07116064	6.443347336	59.69897394	9.265211206
TotA	FBgn0028396	7.927580067	1.587320289	14.26783984	8.988633197
c-cup	FBgn0031367	5.153117446	1.038743247	9.267491645	8.921830946
Lsp1beta	FBgn0002563	5180.744843	1065.082012	9296.407675	8.728349152
CG31313	FBgn0051313	35.05618745	7.22226004	62.89011486	8.70781646
fusl	FBgn0031702	17.65594725	3.646495901	31.66539861	8.683788347
Sr-CIV	FBgn0031547	11.30574587	2.365266571	20.24622517	8.559806922
CG4582	FBgn0039344	5.025817799	1.057054129	8.994581469	8.509102061
CG13641	FBgn0039239	45.02935571	9.563055267	80.49565615	8.417357623
Cyp28a5	FBgn0028940	9.469621111	2.012915194	16.92632703	8.408862467

CG9861	FBgn0034844	13.10574327	2.812650549	23.398836	8.319140819
CG6830	FBgn0037934	4.81594988	1.038743247	8.593156514	8.272647297
Obp99b	FBgn0039685	2851.494376	620.8061273	5082.182625	8.186424717
CG18599	FBgn0038592	10.05158742	2.190829978	17.91234486	8.176054301
Dif	FBgn0011274	1195.247041	262.0502218	2128.44386	8.122274596
CG34165	FBgn0085194	18.96699205	4.18543429	33.74854981	8.063332852
NimB3	FBgn0054003	13.06946511	2.913843613	23.2250866	7.970601612
CG18003	FBgn0061356	22.70386852	5.088874477	40.31886256	7.922943028
Gadd45	FBgn0033153	2563.612599	578.3046795	4548.920519	7.865958344
CG8369	FBgn0040532	48.86325822	11.0457043	86.68081213	7.847468099
Ndg	FBgn0026403	506.6195478	115.7054392	897.5336564	7.757056733
Ir100a	FBgn0039879	10.11310818	2.359106109	17.86711025	7.573678088
CG15369	FBgn0030105	54.43531275	12.90094844	95.96967706	7.438962919
jv	FBgn0263973	6.00360382	1.439866808	10.56734083	7.339109958
Cul2	FBgn0032956	6030.497021	1459.334801	10601.65924	7.264720359
RacGAP84C	FBgn0045843	58.45628655	14.5437803	102.3687928	7.038664686
Tob	FBgn0028397	31.75355246	7.901525026	55.60557989	7.037322504
CG5773	FBgn0034290	52.92989955	13.28627289	92.57352621	6.967606867
Gbp1	FBgn0034199	8.063527983	2.025065615	14.10199035	6.963720211
fon	FBgn0032773	323.1866604	81.29256928	565.0807516	6.951198082
ImpL3	FBgn0001258	1257.211579	317.0930406	2197.330116	6.929606883
Cyp6g1	FBgn0025454	66.98240418	16.97595943	116.9888489	6.891442536
CG18789	FBgn0042127	12.96859798	3.382050242	22.55514572	6.669074706
CG9521	FBgn0030588	80.12592048	20.9782849	139.273556	6.638939108
CG12911	FBgn0033501	24.37874363	6.393438225	42.36404903	6.62617633
CG2736	FBgn0035090	54.1346322	14.55073668	93.71852772	6.440809821
Gbp2	FBgn0034200	34.94566046	9.522955312	60.36836561	6.33924697
CG3448	FBgn0035996	97.20256248	26.63672957	167.7683954	6.298385652
tadr	FBgn0032911	7.759662209	2.190829978	13.32849444	6.083764863
SPH93	FBgn0032638	13.5562294	3.835594682	23.27686412	6.068645424
Osi14	FBgn0040279	483.9937786	137.0089576	830.9785997	6.065140663
CG16743	FBgn0032322	15.18078424	4.317088657	26.04447981	6.032880463
GstD5	FBgn0010041	24.97347762	7.138411436	42.80854381	5.996928615
CG6415	FBgn0032287	15.36615269	4.427920373	26.304385	5.940573178
CG5707	FBgn0026593	6.980933373	2.012915194	11.94895155	5.936142558
dyl	FBgn0066365	30.68100114	8.921957391	52.44004488	5.877639018
Cyp4d1	FBgn0005670	46.61198182	13.67524603	79.54871761	5.816986213
Ppi1	FBgn0051025	15.29792614	4.570758738	26.02509355	5.693823508
CG6910	FBgn0036262	155.1706036	46.38306884	263.9581384	5.690829542
Lsd-1	FBgn0039114	134.452014	40.62560187	228.2784262	5.619078013
CG13457	FBgn0036482	7.366855004	2.227451743	12.50625827	5.614603462
Snap25	FBgn0011288	10.19902814	3.091758397	17.30629789	5.597558304
Ance-4	FBgn0033366	29.18396295	8.964739617	49.40318628	5.510833375
CG8927	FBgn0038405	200.510242	61.66918657	339.3512974	5.502769151
CG32549	FBgn0052549	1481.578624	460.2158232	2502.941425	5.438625312
apolpp	FBgn0087002	18.5388947	5.766594118	31.31119529	5.429755355
CG14082	FBgn0036851	19.27530091	6.042223774	32.50837805	5.380200943
spn-E	FBgn0003483	308.0547219	97.55051304	518.5589307	5.315799113
Cpr67Fa1	FBgn0036108	19.11741955	6.093507727	32.14133137	5.2746846
CG13579	FBgn0035010	48.70767672	15.99710496	81.41824848	5.089561436
chinmo	FBgn0086758	777.1019958	257.3241333	1296.879858	5.03986875
spz4	FBgn0032362	95.21093374	32.48410252	157.9377649	4.8620018
CG12971	FBgn0037078	37.18049324	12.72093031	61.64005617	4.845561973
agt	FBgn0024912	232.2259524	80.03933132	384.4125735	4.802795915

GstE8	FBgn0063492	236.4856049	81.82862932	391.1425804	4.78002117
Drs	FBgn0283461	32.25290521	11.23115439	53.27465603	4.743471079
Lip1	FBgn0023496	25.15418433	8.807476981	41.50089169	4.71200683
Ugt86Di	FBgn0040251	303.3079012	106.4838249	500.1319775	4.696788248
CG8100	FBgn0036410	7.517164565	2.680996183	12.35333295	4.60773985
CG33177	FBgn0053177	24.71657497	8.885335626	40.54781431	4.563453314
CG15347	FBgn0030040	21.12330416	7.613915452	34.63269287	4.548604865
Ady43A	FBgn0026602	3246.809204	1176.784642	5316.833766	4.518102614
DNaseII	FBgn0000477	16.30403478	5.92020806	26.68786151	4.507926282
CG30196	FBgn0050196	96.08700417	34.91256555	157.2614428	4.504436735
GstE3	FBgn0063497	140.322924	51.1371534	229.5086946	4.488100712
Tsp42Ep	FBgn0033137	66.95954616	24.44549118	109.4736012	4.478273738
CG12926	FBgn0033437	23.36559963	8.53298425	38.19821501	4.476536449
CG15673	FBgn0034639	125.3916108	45.80214414	204.9810774	4.475359861
baz	FBgn0000163	13689.58816	5026.650551	22352.52577	4.446803203
Cyp9b2	FBgn0015039	10.75129012	3.962225512	17.54035472	4.426894599
CG8160	FBgn0034011	18.95300408	6.991924378	30.91408378	4.421398475
PGRP-SD	FBgn0035806	12.33135151	4.549936087	20.11276693	4.420450428
CG10126	FBgn0038088	218.638987	80.8051946	356.4727794	4.411508211
CG31279	FBgn0051279	8.04216944	2.978414912	13.10592397	4.400301621
CG9449	FBgn0036875	52.07198125	19.40214861	84.74181389	4.367651006
Cp15	FBgn0000355	28.70493785	10.79592083	46.61395486	4.317737744
CG32165	FBgn0042178	85.24794439	32.11206542	138.3838234	4.30940276
CG13157	FBgn0033732	24.29063767	9.188914818	39.39236052	4.286943703
CG14304	FBgn0038629	818.2417024	310.1365336	1326.346871	4.276654722
Cpr49Ah	FBgn0033731	866.662714	329.7715235	1403.553905	4.256140402
Spat	FBgn0014031	49.84962659	19.0754055	80.62384768	4.226586306
CG3008	FBgn0031643	4139.873489	1593.147372	6686.599606	4.197100485
CG31897	FBgn0051897	11.23768554	4.384171724	18.09119935	4.12648055
CG14275	FBgn0032022	22.20231857	8.67695954	35.72767759	4.117534193
Cyp6a13	FBgn0033304	15.10047811	5.94103071	24.25992551	4.083453982
Corp	FBgn0030028	104.9241607	41.3293382	168.5189832	4.077466289
Idgf1	FBgn0020416	8.896214495	3.505202881	14.28722611	4.076005467
NimB2	FBgn0028543	72.67819783	29.03359437	116.3228013	4.006489854
Hn	FBgn0001208	96.77468126	38.67629155	154.873071	4.004341284
Rbp4	FBgn0010258	41.20356666	16.50775281	65.89938052	3.992026129
mus205	FBgn0002891	2138.493124	856.8712953	3420.114952	3.991398674
CG9664	FBgn0031515	12.9427728	5.187555773	20.69798982	3.989931045
Elal	FBgn0013949	23.68008755	9.5376175	37.8225576	3.965619045
CG4267	FBgn0264979	61.98908473	25.06042278	98.91774668	3.947169908
CG4562	FBgn0038740	106.0676583	42.92492229	169.2103943	3.942008169
Mrp4	FBgn0263316	244.9210099	99.32442034	390.5175994	3.931738017
Ets21C	FBgn0005660	550.2391049	223.7453063	876.7329035	3.918441544
Gr94a	FBgn0041225	31.02845297	12.66599766	49.39090827	3.899488188
CG11658	FBgn0036196	105.0740148	43.12640941	167.0216203	3.872838535
ine	FBgn0011603	41.46954063	17.19414468	65.74493658	3.823681714
CG10916	FBgn0034312	498.9055054	207.5269463	790.2840645	3.808103374
CG6023	FBgn0030912	81.17984264	33.78477966	128.5749056	3.805705022
Root	FBgn0039152	40.20475818	16.99272497	63.41679138	3.731996574
CG12868	FBgn0033945	17.67336033	7.47245193	27.87426873	3.730270732
CG18278	FBgn0033836	160.4474658	68.34402742	252.5509042	3.695288582
CG6026	FBgn0038676	186.7887	80.45333381	293.1240661	3.643404844
CG33493	FBgn0053493	35.04880597	15.1324573	54.96515464	3.632268941
CG5577	FBgn0036759	12.8458734	5.554569338	20.13717746	3.62533551

CG3868	FBgn0036422	18.0964629	7.828281497	28.3646443	3.623355178
CG13954	FBgn0033405	75.67278464	32.75430023	118.5912691	3.620632047
Ku80	FBgn0041627	790.910985	349.7377344	1232.084236	3.522880475
Npc2g	FBgn0039800	39.31343542	17.41329634	61.2135745	3.515335254
Irbp	FBgn0011774	763.6583474	340.2895679	1187.027127	3.488285386
CG42846	FBgn0262035	16.07453941	7.168872739	24.98020609	3.484537528
CG9837	FBgn0037635	17.5572566	7.836953727	27.27755947	3.480632963
Or19b	FBgn0062565	63.87187364	28.57405997	99.16968731	3.470619415
Osi21	FBgn0032359	17.24074967	7.717449781	26.76404956	3.467991412
CG5535	FBgn0036764	817.3098855	368.7621551	1265.857616	3.43272106
CG4945	FBgn0034137	39.66555145	17.92583054	61.40527235	3.425518958
CG6912	FBgn0038290	106.2193888	48.36307838	164.0756993	3.392581796
CG4098	FBgn0036648	235.4022524	107.3071117	363.4973931	3.387449232
hwt	FBgn0264542	119.2612026	54.61754393	183.9048613	3.367138983
CG18547	FBgn0037973	2435.631754	1128.238557	3743.02495	3.317582906
CG13708	FBgn0035577	13.99217392	6.507918635	21.4764292	3.300045744
CG7255	FBgn0036493	24.10564898	11.24192997	36.96936799	3.288525021
spz	FBgn0003495	118.7178892	55.45020586	181.9855726	3.281963877
Cyp18a1	FBgn0010383	102.4051439	47.83605249	156.9742353	3.281504788
CG12224	FBgn0037974	425.3174561	199.588071	651.0468411	3.261952669
CG9270	FBgn0032908	27.46630541	12.9599382	41.97267263	3.238647591
MRP	FBgn0032456	5924.502049	2795.883579	9053.12052	3.238017702
CG6330	FBgn0039464	452.5846306	215.9760146	689.1932466	3.191063822
CG7470	FBgn0037146	136.2038721	65.04908697	207.3586572	3.187725868
Cpr76Bc	FBgn0036880	39.01852787	18.6461619	59.39089384	3.185153821
CG14907	FBgn0038455	139.8414728	66.86753884	212.8154068	3.18264154
Lig4	FBgn0030506	392.4622468	188.1811052	596.7433883	3.171112146
amd	FBgn0000075	1497.256558	720.1574214	2274.355694	3.158136855
CG30090	FBgn0050090	13.64083188	6.583673932	20.69798982	3.143835803
Qtzl	FBgn0051864	131.2466473	63.77771329	198.7155812	3.115752682
CG10445	FBgn0037531	273.79019	133.2142797	414.3661002	3.110523145
ect	FBgn0000451	80.2647188	39.40546564	121.123972	3.073786085
Aldh	FBgn0012036	1304.972265	643.7261307	1966.2184	3.054433099
Spn47C	FBgn0033574	68.89314814	34.13120849	103.6550878	3.036959204
AQP	FBgn0033807	17.60287359	8.744042607	26.46170457	3.026255219
Thor	FBgn0261560	203.5491565	101.5137724	305.5845406	3.010276669
Idgf3	FBgn0020414	67.20581966	33.73811083	100.6735285	2.983970532
Ttc19	FBgn0032744	309.3407551	156.2398277	462.4416824	2.95981946
gd	FBgn0000808	17.02320003	8.600067317	25.44633274	2.958852739
GstD9	FBgn0038020	448.7091289	228.9188819	668.4993759	2.92024568
CG14990	FBgn0035496	20.86589017	10.70202515	31.02975519	2.899428355
CG31029	FBgn0051029	102.6562584	52.91518523	152.3973315	2.8800302
CG1850	FBgn0033154	18.37060024	9.52546708	27.21573341	2.857154739
CG8129	FBgn0037684	35.15877407	18.26950969	52.04803846	2.848901768
CG7506	FBgn0035805	267.9220037	139.3675057	396.4765018	2.844827422
CG8852	FBgn0031548	35.156093	18.39266233	51.91952368	2.822838954
GstE5	FBgn0063495	95.62118584	50.04227316	141.2000985	2.821616397
Traf4	FBgn0026319	1014.316634	531.6053277	1497.02794	2.816051425
obe	FBgn0038344	3006.281818	1595.720461	4416.843176	2.767930401
unc-13	FBgn0025726	558.416072	296.5778	820.254344	2.765730759
CG12264	FBgn0032393	1842.116867	980.3521046	2703.881629	2.758071938
CG31549	FBgn0051549	1587.206362	844.8205493	2329.592176	2.757499421
upd1	FBgn0004956	361.6003186	193.7961887	529.4044484	2.731758823
Irc	FBgn0038465	45.46767734	24.41908699	66.51626769	2.723945729

CG34446	FBgn0085475	17.7104211	9.571727497	25.8491147	2.700569433
ver	FBgn0262524	222.6697829	121.6216933	323.7178726	2.661678718
CG1299	FBgn0035501	928.0638135	508.6832466	1347.44438	2.648886885
CG10433	FBgn0034638	24.17475121	13.32695177	35.02255065	2.627949081
CG18596	FBgn0038953	705.1657403	388.8136506	1021.51783	2.627268431
Men	FBgn0002719	1774.771514	986.9518551	2562.591173	2.596470294
sPLA2	FBgn0033170	54.44441712	30.41140165	78.47743259	2.580526655
CG32668	FBgn0052668	18.26013754	10.20934718	26.3109279	2.577141068
lama	FBgn0016031	1601.266706	897.9645753	2304.568836	2.566436249
rev7	FBgn0037345	32.65901799	18.33504741	46.98298856	2.562468889
Reg-2	FBgn0016715	120.9445212	68.14649432	173.7425481	2.549544915
CG7130	FBgn0037151	316.6373928	179.2955317	453.979254	2.532016553
CG42365	FBgn0259711	88.49656129	50.61442254	126.3787	2.496891078
l(2)k05911	FBgn0051728	1936.192215	1111.975903	2760.408526	2.482435561
Ddc	FBgn0000422	352.4674849	202.9431382	501.9918316	2.473559028
CG1358	FBgn0033196	41.85691712	24.16427998	59.54955426	2.464362866
CG31793	FBgn0051793	712.1068888	411.3104778	1012.9033	2.462624597
CG2017	FBgn0037391	1519.827258	878.830001	2160.824515	2.458751422
CG14367	FBgn0038170	20.02190297	11.57848223	28.4653237	2.458467625
CG3036	FBgn0031645	617.8216548	357.9917394	877.6515702	2.451597268
CG8563	FBgn0035777	48.24406415	28.02376708	68.46436122	2.44308201
CG6142	FBgn0039415	99.35273637	57.84649174	140.858981	2.435047948
teq	FBgn0023479	182.8736571	106.7905413	258.9567729	2.424903645
CG1882	FBgn0033226	897.4467578	530.7450572	1264.148458	2.381837459
Archease	FBgn0038893	328.5862698	194.3376389	462.8349007	2.381601955
mre11	FBgn0020270	2213.406686	1309.508383	3117.30499	2.380515491
Pdp1	FBgn0016694	300.76249	178.765095	422.759885	2.364890556
CG14079	FBgn0036849	22.37727834	13.41599441	31.33856227	2.335910505
CG10189	FBgn0032793	297.1931997	178.1906718	416.1957275	2.335676292
CG31337	FBgn0051337	28.06354657	16.8417933	39.28529984	2.332607884
Irbp18	FBgn0036126	34.0916791	20.46285143	47.72050676	2.332055575
CG3168	FBgn0029896	437.097265	262.5837809	611.6107491	2.32920231
Eglp4	FBgn0034885	25.60359796	15.38977607	35.81741984	2.327351592
Mocs1	FBgn0263241	831.5327418	502.4524355	1160.613048	2.309896352
GstE1	FBgn0034335	428.5020732	259.3956298	597.6085165	2.303849594
CG13868	FBgn0034501	153.6608348	93.03193726	214.2897324	2.303399657
CG7778	FBgn0032025	30.9411937	18.74986674	43.13252067	2.300417452
mthl2	FBgn0035623	31.41598014	19.16096996	43.67099033	2.279163864
Ptp52F	FBgn0034085	27.16752248	16.5871568	37.74788817	2.275729869
rad50	FBgn0034728	1392.955434	850.7956962	1935.115172	2.274476917
ftz-f1	FBgn0001078	563.1148366	344.8562486	781.3734246	2.265794596
eIF6	FBgn0034915	3467.79213	2136.519498	4799.064762	2.246206865
CG42553	FBgn0260755	323.382689	199.3122035	447.4531744	2.244986341
CG18563	FBgn0032639	25.81043845	15.93835311	35.68252379	2.238783614
Socs36E	FBgn0041184	5378.533198	3337.547932	7419.518464	2.223044767
p53	FBgn0039044	274.6904475	170.472664	378.9082309	2.222692026
glob1	FBgn0027657	172.1810124	106.9653654	237.3966595	2.219378755
CG32698	FBgn0052698	106.4932424	66.53219092	146.4542939	2.201254639
f	FBgn0262111	183.0918898	114.4621391	251.7216405	2.199169459
CG18094	FBgn0032791	55.51671814	34.7090425	76.32439379	2.19897722
CG8157	FBgn0034010	73.38543696	46.0170217	100.7538522	2.189490943
Cyp9f2	FBgn0038037	94.8173581	59.47700267	130.1577135	2.188370424
NimC1	FBgn0259896	134.12059	84.28119711	183.9599829	2.182693046
NKCC	FBgn0051547	108.5086552	68.23382112	148.7834893	2.180494759

CG7069	FBgn0038952	44.72777079	28.15947856	61.29606302	2.17674709
CG9279	FBgn0036882	363.8543446	229.5511223	498.1575669	2.170137797
Ent3	FBgn0036319	27.96617148	17.64517735	38.28716562	2.169837393
Bin1	FBgn0024491	1666.016411	1051.721359	2280.311463	2.168170726
trbl	FBgn0028978	3788.303291	2391.540686	5185.065895	2.168086006
CG13437	FBgn0034541	27.5477745	17.44530299	37.65024602	2.158188141
CG32687	FBgn0052687	58.46994944	37.02764023	79.91225864	2.158178543
CG14984	FBgn0035480	644.5944127	408.5887501	880.6000753	2.155223498
Xrp1	FBgn0261113	28028.77382	17820.45761	38237.09003	2.145685081
IscU	FBgn0037637	1329.07426	845.8272038	1812.321317	2.142661419
mus201	FBgn0002887	650.2968566	413.9385308	886.6551823	2.14199722
CG5080	FBgn0031313	36.20726878	23.08178808	49.33274948	2.137301898
CG12268	FBgn0039131	1685.512207	1077.417593	2293.606821	2.128800231
CG30046	FBgn0050046	92.27224724	58.9958497	125.5486448	2.12809283
Gclc	FBgn0040319	1063.496622	682.9888439	1444.004401	2.114243027
yellow-b	FBgn0032601	441.0669868	283.4969513	598.6370223	2.111617143
Mal-B2	FBgn0032382	52.51653912	33.93223314	71.1008451	2.095377714
CG1146	FBgn0035346	1538.476693	1000.088805	2076.864581	2.076680162
CG7131	FBgn0038598	101.7126012	66.26791576	137.1572866	2.069738953
CG31633	FBgn0051633	56.40216401	36.76935504	76.03497298	2.067889766
CG3253	FBgn0041706	93.3091859	60.84418395	125.7741878	2.067152186
Jheh3	FBgn0034406	34.36077071	22.41718529	46.30435614	2.065574047
Shal	FBgn0005564	68.82351864	44.99373656	92.65330073	2.05924886
tut	FBgn0052364	110.4325038	72.41594772	148.4490598	2.049949832
pn	FBgn0003116	92.00971197	60.37872701	123.6406969	2.047752628
Dgp-1	FBgn0027836	2688.752074	1765.991381	3611.512767	2.045034197
eIF2D	FBgn0041588	1357.156339	891.6765748	1822.636103	2.044055159
CG9945	FBgn0034527	807.7008942	530.7546494	1084.647139	2.04359423
CG1582	FBgn0030246	2639.593126	1737.067934	3542.118318	2.039136321
CG18213	FBgn0038470	41.73915303	27.48637404	55.99193202	2.037079607
CG8060	FBgn0034113	486.2247541	320.2191678	652.2303403	2.036824793
CG15097	FBgn0034396	129.6778645	85.44565126	173.9100778	2.035329771
CG7054	FBgn0038972	99.67053094	65.80026714	133.5407947	2.029487121
CG2121	FBgn0033289	47.06281407	31.10378348	63.02184467	2.026179378
CG5281	FBgn0037902	42.63292342	28.23620028	57.02964656	2.019735162
wfs1	FBgn0039003	1019.315125	676.4152635	1362.214986	2.013873813
CG6654	FBgn0038301	1322.757215	878.2461514	1767.268279	2.012269882
egr	FBgn0033483	485.0312425	322.0625669	647.9999181	2.012031154
CngB	FBgn0266346	49.28858038	32.80558418	65.77157657	2.004889662
CG10621	FBgn0032726	46.78122847	31.18552874	62.3769282	2.000188252
CG18636	FBgn0032551	2.992464111	0	5.984928222	Inf
Spn88Eb	FBgn0038299	4.274679855	0	8.549359711	Inf
Gr59c	FBgn0041235	2.825136396	0	5.650272793	Inf
Gr59d	FBgn0041236	2.686888079	0	5.373776158	Inf
CG31664	FBgn0051664	13.25777496	0	26.51554992	Inf
CG32695	FBgn0052695	3.3167075	0	6.633415	Inf

B. Downregulated gene list

Gene Symbol	id	baseMean	baseMean control	baseMeanB dNKAP RNAi	foldChange
CG13032	FBgn0036652	22.50931876	44.6416231	0.377014417	0.008445356

Cyp6a17	FBgn0015714	55.12101648	109.1109897	1.131043251	0.010365988
CG30059	FBgn0260475	26.79858413	52.86754997	0.729618296	0.013800872
CG15236	FBgn0033108	24.36946212	47.63229152	1.106632713	0.023232825
y	FBgn0004034	6.909017634	13.47189348	0.346141791	0.025693626
CG30485	FBgn0050485	11.08029546	21.40656208	0.754028834	0.035224191
sand	FBgn0033257	203.1652731	390.3499458	15.98060035	0.040939164
CG18586	FBgn0035642	3.842213793	7.338285795	0.346141791	0.047169298
CG14957	FBgn0035412	56.44994095	107.8119401	5.087941805	0.047192749
CG31683	FBgn0051683	32.67926696	62.13125366	3.227280259	0.051942944
CG4563	FBgn0035006	6.509608606	12.32693363	0.692283581	0.056160242
Cyp6t3	FBgn0033697	118.9490552	215.1785531	22.7195574	0.105584674
CG8564	FBgn0035776	15.74663829	27.4696085	4.023668081	0.146477081
CG14237	FBgn0039428	10.2500277	17.68066219	2.819393215	0.15946197
CG14238	FBgn0039429	22.21062785	37.17533163	7.245924066	0.194912157
kat-60L1	FBgn0037375	15.38279148	25.12823435	5.637348616	0.224343204
Cpr66Cb	FBgn0035875	19.53342642	31.86974984	7.19710299	0.225828663
Toll-9	FBgn0036978	15.83737934	25.61013675	6.064621924	0.236805527
nrm	FBgn0262509	33.34189951	53.62677695	13.05702208	0.243479523
Cpr47Ee	FBgn0033602	25.93603004	41.07201942	10.80004066	0.262953729
Pten	FBgn0026379	46.19151463	73.04375825	19.33927101	0.264762814
CG2930	FBgn0028491	85.49571919	133.9947842	36.99665415	0.276105181
Culd	FBgn0035880	1908.465724	2961.403776	855.5276719	0.288892612
CG34334	FBgn0263829	26.27346444	39.94805273	12.59887614	0.315381484
Hr3	FBgn0000448	75.61960915	114.0340324	37.20518588	0.32626388
CG4570	FBgn0037844	35.25845104	53.05121679	17.46568529	0.329223086
sm	FBgn0003435	307.3437514	461.7348351	152.9526677	0.331256505
Spn100A	FBgn0039795	2594.587371	3889.412239	1299.762503	0.334179671
CG6357	FBgn0033875	21.94475161	32.64136516	11.24813805	0.34459766
Tsp42Ei	FBgn0033130	49.22487425	72.8899738	25.5597747	0.350662421
ND-MLRQ	FBgn0052230	21.57584693	31.78150312	11.37019074	0.357761264
CG30054	FBgn0050054	21.55276561	31.63769833	11.46783289	0.362473678
Lcp2	FBgn0002533	22.27924918	32.66339215	11.8951062	0.364172409
CG31559	FBgn0051559	192.9353334	282.1515234	103.7191433	0.367600862
CG9961	FBgn0031451	26.93497184	39.15544426	14.71449942	0.375797024
CG8483	FBgn0038126	400.4167015	580.2846851	220.548718	0.380069858
prom	FBgn0259210	41.0596993	59.10754475	23.01185384	0.389321768
nAChRalpha1	FBgn0000036	31.97637741	45.77037541	18.18237941	0.397252136
Rpb11	FBgn0032634	33.18368855	47.33835098	19.02902612	0.401979066
CG1139	FBgn0035300	45.05527532	64.25345522	25.85709541	0.402423423
mt:CoII	FBgn0013675	280.3264619	399.5939537	161.0589701	0.403056574
CG3355	FBgn0031619	112.5107701	159.2167647	65.80477563	0.413303057
CG13023	FBgn0036677	276.8905018	388.4575071	165.3234965	0.425589655
CG13082	FBgn0032803	478.2883682	670.5324375	286.0442989	0.426592783
CG8420	FBgn0037664	551.7080369	771.7018022	331.7142717	0.429847735
CG10257	FBgn0033985	34.39397624	48.0641143	20.72383818	0.431170708
Zyx	FBgn0011642	60.46776644	84.48676692	36.44876597	0.431413904
CG7637	FBgn0033548	34.85450176	48.53635712	21.17264639	0.436222404
CG30440	FBgn0050440	85.18641315	118.4446757	51.92815056	0.438416925
dib	FBgn0000449	63.73943542	88.06497541	39.41389543	0.44755472
CG30427	FBgn0043792	35.90119531	49.27461187	22.52777874	0.457188355
Haspin	FBgn0046706	40.30501508	55.17382678	25.43620338	0.461019379
ImpE2	FBgn0001254	39354.61139	53805.67336	24903.54941	0.462842445
fs(1)Yb	FBgn0000928	31.66823068	43.26900986	20.0674515	0.463783469
CG7737	FBgn0033584	33.04299374	45.10862539	20.97736209	0.465041041

Sans	FBgn0033785	1082.192268	1477.125182	687.2593537	0.465268186
CG15756	FBgn0030493	229.7693892	311.5271694	148.011609	0.475116213
Nipped-A	FBgn0053554	173.3376133	234.8492153	111.8260114	0.47616089
CG30383	FBgn0050383	35.14494989	47.59339591	22.69650387	0.476883472
CG6610	FBgn0035675	48.50636051	65.66131538	31.35140563	0.477471483
Fis1	FBgn0039969	31.50790214	42.64313218	20.37267211	0.477748023
Gprk1	FBgn0260798	70.24829662	94.89233976	45.60425347	0.480589409
dy	FBgn0004511	134.6492463	181.774685	87.52380773	0.481496132
CG5065	FBgn0034145	261.6835585	352.8285371	170.5385799	0.483346901
m	FBgn0002577	144.4071906	194.3209198	94.49346144	0.486275289
Slip1	FBgn0024728	85.89771819	115.4565548	56.33888161	0.487966073
Spn31A	FBgn0032178	38.92026093	52.05315242	25.78736944	0.495404567
CG2187	FBgn0017448	6.311124506	12.62224901	0	0
Cyp4p2	FBgn0033395	4.665876591	9.331753182	0	0
Obp58b	FBgn0034768	3.273526084	6.547052167	0	0
CG44098	FBgn0264907	4.760221772	9.520443544	0	0

Table S3 GO analysis of differentially expressed genes in *dNKAP* knockdown tissues as compared to the control

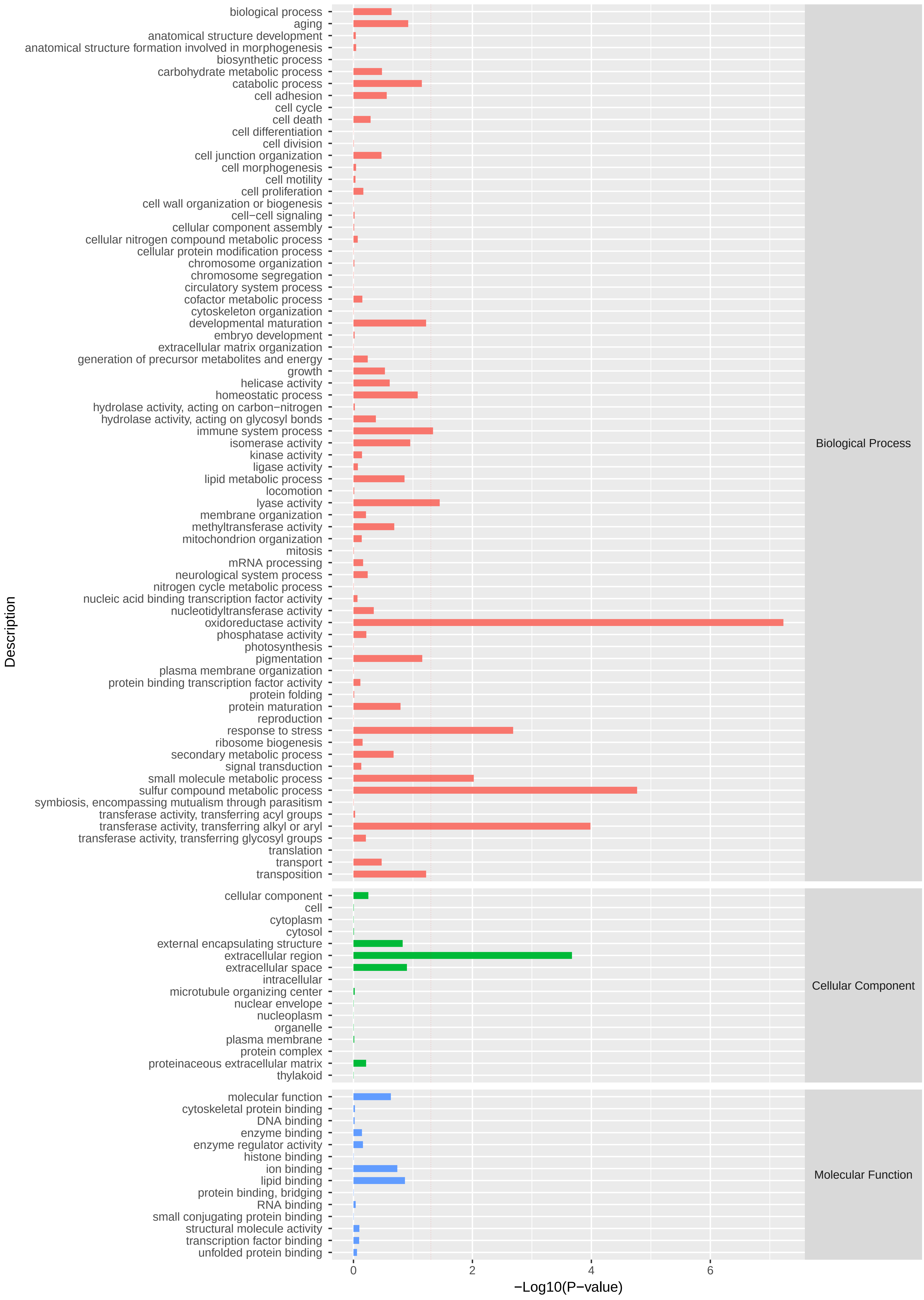

biological process
aging
anatomical structure development
anatomical structure formation involved in morphogenesis
biosynthetic process
carbohydrate metabolic process
catabolic process
cell adhesion
cell cycle
cell death
cell differentiation
cell division
cell junction organization
cell morphogenesis
cell motility
cell proliferation
cell wall organization or biogenesis
cell−cell signaling
cellular component assembly
cellular nitrogen compound metabolic process
cellular protein modification process
chromosome organization
chromosome segregation
circulatory system process
cofactor metabolic process
cytoskeleton organization
developmental maturation
embryo development
extracellular matrix organization
generation of precursor metabolites and energy
growth
helicase activity
homeostatic process
hydrolase activity, acting on carbon−nitrogen
hydrolase activity, acting on glycosyl bonds
immune system process
isomerase activity
kinase activity
ligase activity
lipid metabolic process
locomotion
lyase activity
membrane organization
methyltransferase activity
mitochondrion organization
mitosis
mRNA processing
neurological system process
nitrogen cycle metabolic process
nucleic acid binding transcription factor activity
nucleotidyltransferase activity
oxidoreductase activity
phosphatase activity
photosynthesis
pigmentation
plasma membrane organization
protein binding transcription factor activity
protein folding
protein maturation
reproduction
response to stress
ribosome biogenesis
secondary metabolic process
signal transduction
small molecule metabolic process
sulfur compound metabolic process
symbiosis, encompassing mutualism through parasitism
transferase activity, transferring acyl groups
transferase activity, transferring alkyl or aryl
transferase activity, transferring glycosyl groups
translation
transport
transposition
Biological Process
cellular component
cell
cytoplasm
cytosol
external encapsulating structure
extracellular region
extracellular space
intracellular
microtubule organizing center
nuclear envelope
nucleoplasm
organelle
plasma membrane
protein complex
proteinaceous extracellular matrix
thylakoid
Cellular Component
molecular function
cytoskeletal protein binding
DNA binding
enzyme binding
enzyme regulator activity
histone binding
ion binding
lipid binding
protein binding, bridging
RNA binding
small conjugating protein binding
structural molecule activity
transcription factor binding
unfolded protein binding
Molecular Function
Description
0
2
4
6
−Log10(P−value)

Table S4 Primers used for RT-qPCR analysis

Primer list	Sequence (5’-3’)
Rp49-F	GCTAAGCTGTCGCACAAA
Rp49-R	TCCGGTGGGCAGCATGTG
dNKAP-F	AGATGTTTGGCTGGAAAAGA
dNKAP-R	TGCCGAAGTCCTTTTGATTA
p53-F	CTTCAAGTTCGTCTGCC
p53-R	CGTCTTGGATGCGATC
Xrp1-F	GACTGATGCTCCAGATC
Xrp1-R	CTGCTGATGGAAGTGTTG
mre11-F	GTTCAGGATGTGGTGCA
mre11-R	CACTAACTCCTCCACG
Cyp6d2-F	ATTCCTCAATCGAGAGTGCACCGA
Cyp6d2-R	TGCATGCCAAAGAGCGAGATCAGA
Gclc-F	GTGACATATTGAAATGGGG
Gclc-R	TTCCTTCTCATTGAGCTGTGC
MRP-F	AATCGAAAGTATGGCGTGCAG
MRP-R	GGGGAATCGACAGCACAGT
Socs36E-F	CAGTCAGCAATATGTTGTCG
Socs36E-R	ACTTGCAGCATCGTCGCTTC
Gadd45-F	CTGGAGGCCTTTTGCTACGA
Gadd45-R	GTCGACTAGCTGGTTCTCGG
Upd2-F	GTACAAGTTCCTGCCGAACATG
Upd2-R	GATCTCGCAGAGCATATAGC
Upd3-F	GAGCACCAAGACTCTGGACA
Upd3-R	CCAGTGCAACTTGATGTTGC
Dif-F	GTGTGCAACTACGATG
Dif-R	GATCTTGTCGATCTGGTC
Gd-F	GCTACACATCCAACGAG
Gd-R	GACGACAGCTTCTGATC
Lig4-F	CATCAGATGCTGGACAC
Lig4-R	CAGCGCTGGTAGATATC
Dy-F	CAGATGCAGTTGCCAC
Dy-R	GTCCCTTGTAGAACTGG
MMP1-F	TACAGATTCCATGACGCCCG
MMP1-R	TTAAAGCCTGTGGACGAGGG
Puc-F	AGCCCTCCTACCGAAAATCA
Puc-R	CCGCTTGTTGGGATAGTCCT
Chinmo-F	TGTACAAAGGCGAGGTCCAC
Chinmo-R	CCCATGTGTTGTTGTGCCTG
ImpL3-F	GTCCGCTTGAAGTGCAAACA
ImpL3-R	GACATCGATGAGGCACACCT

Ets21C-F	GAAGGAACTCCAGCGACTCC
Ets21C-R	GTTCAGCAGCTGGTACGGAT
Dilp8-F	GCACCACCATCTGAATCGAC
Dilp8-R	AAAGATGTCCAGTTCGCTGA
ImpE2-F	AGAGACTGTCGTTGTGCCTG
ImpE2-R	ATCATCCGCCTGGACTTGTG
Socs36E-F2	GCGGTCACAAGTTCAGCTTCG
Socs36E-R2	TCTGTGCAGGGGTATCGTCA

Table S5 Lists of each alternative splicing event altered in *dNKAP* knockdown wing discs
